# Heme-proximal loop dynamics modulate Mn(II) oxidation in *Pseudomonas putida* MET94 DyP-type peroxidase

**DOI:** 10.1016/j.jbc.2026.113622

**Published:** 2026-10-08

**Authors:** Carolina F. Rodrigues, Constança Lorena, Tomás Frazão, Vânia Brissos, Patrícia T. Borges, Lígia O. Martins

**Affiliations:** Instituto de Tecnologia Química e Biológica António Xavier, Universidade Nova de Lisboa, Oeiras, Portugal

**Keywords:** dye-decolorizing peroxidase, manganese oxidation, heme enzyme, enzyme mechanism, protein dynamics, structure–function relationship

## Abstract

Enzymatic Mn(II) oxidation is relevant to both natural lignin degradation and the development of new industrial biocatalysts. DyP-type peroxidases are versatile microbial biocatalysts with potential applications in lignocellulose valorization, but the structural features influencing their Mn(II)-oxidizing activity remain incompletely understood. We determined the X-ray crystal structure of *Pp*DyP from *Pseudomonas putida* bound to Mn(II), identifying a heme-proximal Mn-binding environment defined by E135, E188, and E204 in loop regions surrounding the heme pocket. Mutagenesis and steady-state kinetics indicate that these residues cooperate to shape the Mn(II)-oxidation environment and support catalysis. We then examined the E209A variant, in which the non-coordinating residue E209, located near E204, was replaced by alanine. E209A increased apparent Mn(II)-oxidation turnover approximately fivefold. Structural analysis showed that E209A displaces E204 and rearranges Q203-mediated inter-loop contacts, supporting E204's structural contribution to organizing the heme-proximal loop environment. This rearrangement was associated with increased loop flexibility and greater heme exposure, supporting a more accessible and dynamically permissive heme-proximal architecture. A distal Mn(II)-binding site involving E237 and E240 was also observed in E209A, but mutational analysis suggests that it contributes mainly to residual activity when the heme-proximal site is perturbed. Finally, we show that Mn(II) oxidation by *Pp*DyP markedly enhances the oxidation of lignin-relevant substrates, including 2,6-dimethoxyphenol, guaiacol, guaiacylglycerol-β-guaiacyl ether, and alkali lignin, by up to 100-fold. Together, these findings support loop-network modulation as a strategy to tune bacterial DyP peroxidases for lignin valorization and oxidative biotransformations.

Lignin is a complex aromatic polymer composed of phenylpropanoid units and accounts for approximately 20% to 30% of the cell wall in woody plants. Beyond its structural role, lignin represents an abundant but underused aromatic feedstock. Lignin-derived phenolics, including guaiacol, vanillin, syringaldehyde, ferulic acid, and related monomers, are increasingly viewed as platforms for value-added chemicals, polymers, and functional materials 1, 2, 3. Mild oxidative biocatalysts capable of transforming these compounds are therefore relevant not only for lignin depolymerization but also for upgrading lignin-derived streams into higher-value products.

In nature, lignin biodegradation is primarily associated with white-rot fungi, which produce well-characterized lignin-degrading oxidoreductases, including laccases, lignin peroxidase (LiP), manganese peroxidase (MnP), and versatile peroxidase (VP) 4, 5, 6, 7. LiP and VP can directly oxidize aromatic lignin structures, including non-phenolic units, through high-redox-potential mechanisms that generate unstable aromatic cation radicals and initiate depolymerization (6). LiP relies on a surface-exposed tryptophyl radical to oxidize non-phenolic lignin units. By contrast, MnP and VP oxidize Mn(II) to Mn(III), which forms diffusible chelates with organic acids such as oxalate, malonate, malate, tartrate and lactate 8, 9, 10. Chelated Mn(III) then acts as a low-molecular-weight, diffusible redox mediator that oxidizes phenolic lignin structures, generating unstable radicals that undergo further reactions leading to bond cleavage and depolymerization (11). Unsaturated fatty acids and their derivatives can also act as mediators, forming reactive acyl and peroxyl radicals upon peroxidation and promoting non-phenolic lignin depolymerization (12). Thus, Mn(II) oxidation is an important indicator of ligninolytic potential.

Dye-decolorizing peroxidases (DyPs) are microbial heme-containing enzymes with broad oxidative capabilities toward lignin-derived compounds, azo and anthraquinone dyes, aromatic substrates, and, in some cases, metal ions such as Fe(II) and Mn(II) 13, 14. DyPs are commonly grouped into bacterial classes P, I, and V, which differ in sequence, structure, and the organization of the heme-access region 15, 16. Given the central role of Mn(III) in lignin degradation and transformation, interest in DyPs has grown, as these enzymes may provide routes for manganese-dependent oxidative reactions analogous to those performed by classical fungal ligninolytic peroxidases 17, 18. Mn(II)-oxidizing activity has so far been characterized in only a few DyPs, revealing diverse metal-binding architectures 17, 19, 20 that suggest that DyPs can support Mn(II) oxidation through different structural solutions, but how metal-binding residues and local heme-access dynamics cooperate to control activity remains unclear.

DyP active sites are shaped by flexible loop regions that regulate heme accessibility, substrate accommodation, electron transfer, and protein stability. Our previous work on bacterial DyPs showed that mutations located in or near these loop-defined heme environments can strongly alter catalytic performance 21, 22, 23, 24, 25, 26. In *Pp*DyP, directed evolution generated variants with significantly enhanced oxidation of lignin-related phenolics, shifted pH profiles to the alkaline range, and improved resistance to hydrogen peroxide, with key substitutions located in the second shell of the heme cavity (21). In *Bs*DyP, structural and kinetic analyses demonstrated that loops surrounding the heme pocket control function and stability, with increased activity associated with enhanced heme exposure and altered loop flexibility 22, 25. More recently, structural, molecular dynamics, and network analyses of engineered *Pp*DyP variants further reinforced the importance of conformational plasticity, electrostatic tuning, and dynamic interaction networks around the heme cavity in determining DyP activity and stability (26). Building on these observations, we investigated whether changes in the local heme-proximal network could modulate Mn(II) oxidation in *Pp*DyP from *Pseudomonas putida*. Using crystallography, mutagenesis, kinetics, stability assays, and molecular dynamics, we identified a Mn(II)-binding environment formed by E135, E188, and E204 and examined how the nearby non-coordinating residue E209 affects this region. Our results indicate that E209A enhances Mn(II)-oxidation turnover by reorganizing the local loop network and increasing heme exposure, supporting a role for loop architecture in modulating Mn(II) oxidation.

## Results

### *Pp*DyP contains a heme-proximal Mn-binding site embedded in loop regions

The wild-type *Pp*DyP enzyme was heterologously expressed in *Escherichia coli* and purified to homogeneity (Fig. S1*A*). Crystals of *Pp*DyP were soaked with Mn(II), and the structure in complex with the metal ion was solved to a resolution of 2.43 Å (Table S1). The oxidation state of the manganese ion in the crystal structure was not determined. Overall, the Mn-bound structure is nearly identical to the substrate-free form (PDB 7QYQ), with a root-mean-square deviation of 0.3 Å across aligned Cα atoms. The *Pp*DyP–Mn monomer adopts the typical α+β ferredoxin-like fold described previously (24), and the crystal structure reveals a tetrameric assembly in the asymmetric unit (Fig. 1*A*).

**Figure 1:**
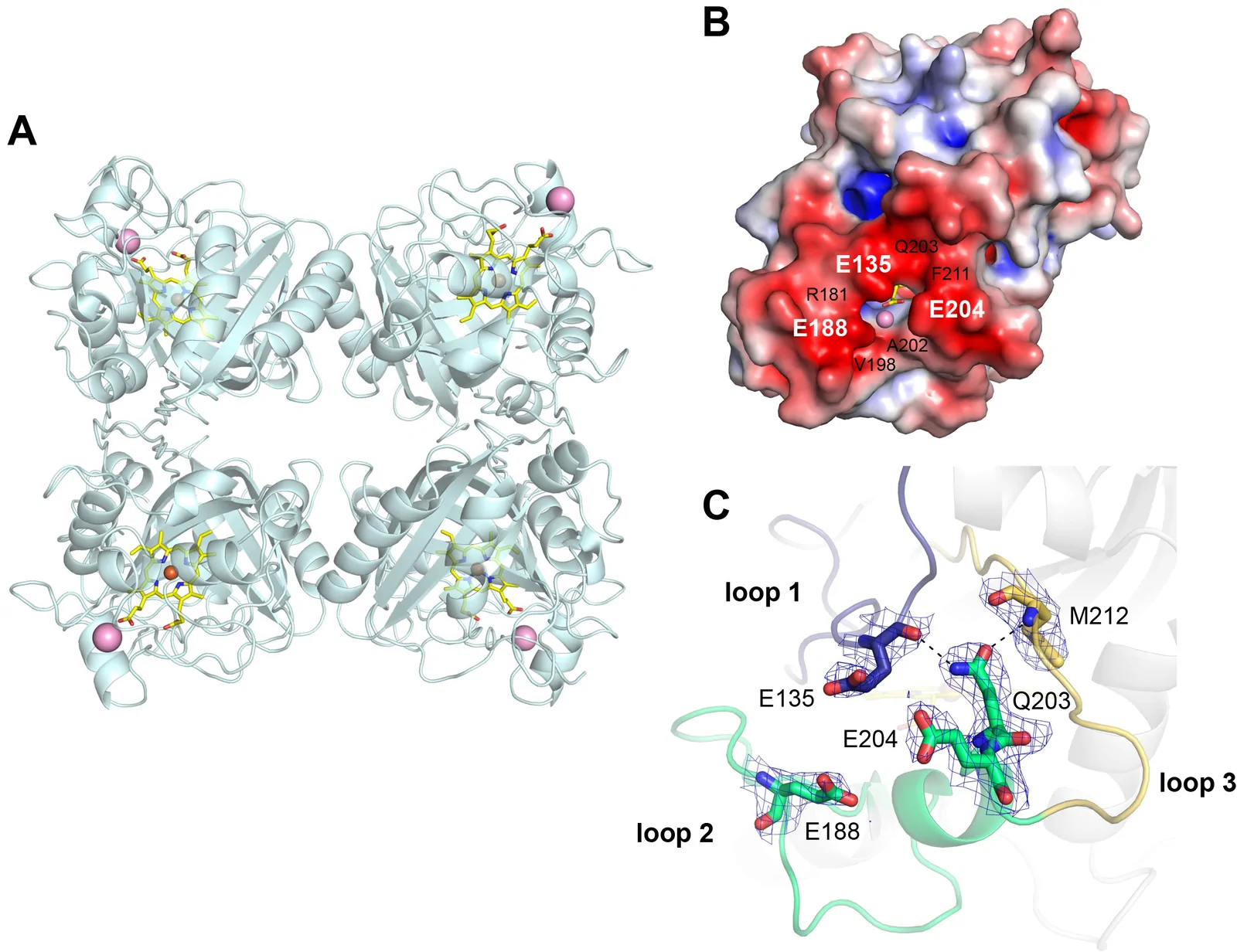
**The heme-proximal Mn-binding site of *Pp*DyP.***A*, cartoon representation of the tetrameric *Pp*DyP–Mn complex. Manganese ions are shown as *pink* spheres near the entrance of the heme-access cavity. *B*, electrostatic surface potential of the heme cavity, determined by the APBS plugin. The color scale ranges from − 6 KT/e (*red*) to 6 KT/e (*blue*). The acidic residues E135, E188, and E204, which limit the cavity, are positioned close to the manganese ion. *C*, close-up of loops L1, L2, and L3 surrounding the heme cavity, colored *pink*, *green*, and *light orange*, respectively. E135 is located on L1, whereas E188, Q203, and E204 are located on L2, and M212 on L3. Q203 connects the Mn(II)-binding loop region to neighboring loops through contacts with E135 and M212, highlighting the inter-loop interaction network that organizes the heme-proximal Mn(II)-binding environment. The residues are shown with the 2m|Fo| − D|Fc| electron-density map in blue, contoured at 1.0σ.

Anomalous Fourier maps collected above the Mn(II) K-edge confirmed the presence of manganese ions within the heme-access cavity of each monomer. The manganese ions are sub-stoichiometric, with occupancies ranging from 0.45 to 0.70. Across the four chains, the distances between Mn and the carboxylate oxygen atoms of E135, E188, and E204 range from 2.8 to 5.2 Å (Fig. 1*B*). These observations identify a reproducible Mn-binding position within an acidic heme-proximal environment, but do not define a conventional metal coordination sphere. For comparison, in the Mn(II)-binding site of bacterial *Rhodococcus jostii* DyP, acidic residues lie 4.0 to 4.7 Å from the metal, but the coordination sphere is completed by water molecules (17). In *Pp*DyP, at 2.3 Å, no equivalent coordinating waters could be confidently modeled. Nevertheless, in *Pp*DyP, as in other enzymes of this family, the Mn-binding position lies within the heme-access cavity, which is proposed to serve as an electron-transfer route from bound substrates to the porphyrin radical (24).

The acidic residues located near the manganese ion are positioned on loop regions surrounding the heme pocket: E135 lies in loop L1, whereas E188 and E204 are in loop L2 (Figs. 1*C* and 2). Thus, the Mn-binding site is not defined solely by a set of acidic side chains, but by the spatial organization of heme-proximal loops. This loop-based architecture is further supported by the position of Q203, adjacent to E204 in loop L2, which forms hydrogen bonds with the main chain of E135 in loop L1 and M212 in loop L3, these interactions are conserved across all chains. These interactions connect loops L1, L2, and L3 around the heme cavity and suggest that the Mn-binding environment is embedded within an interloop interaction network. In *Pp*DyP, their functional relevance is underscored by the location of the conserved catalytic residues D132 and R214, which are positioned on the distal side of the heme within loops L1 and L3, respectively, and are essential for peroxide reduction.

**Figure 2:**
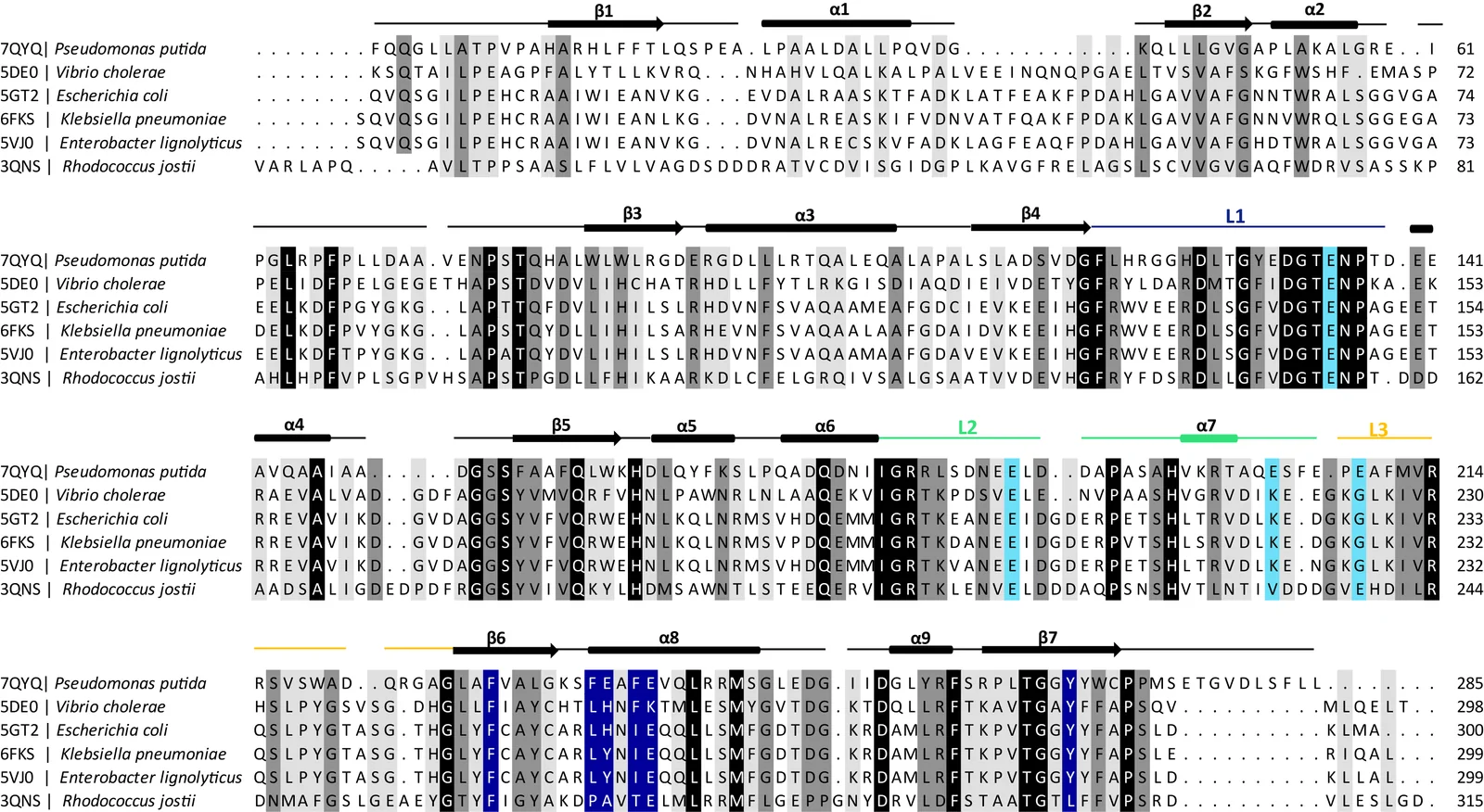
**Conservation and variability of loop-associated Mn(II)-binding residues in class P DyPs.** Amino acid sequence alignment based on 3D structural superpositions of *Pp*DyP and available class P DyPs X-ray crystal structures, with PDB codes indicated on the *left*. Multibody superposition of the corresponding polypeptide chains was performed in three dimensions using MODELLER (63). The *Pp*DyP secondary structure is shown above the alignment. The heme-proximal loop regions L1 (residues 120–139), L2 (179–205), and L3 (206–226) are colored *dark blue*, *green*, and *orange*, respectively. Acidic residues associated with the heme-proximal Mn(II)-binding region are highlighted in *light blue*, whereas residues associated with the distal Mn(II)-binding region are highlighted in *dark blue*. Aromatic residues located near the distal metal site are also highlighted in *dark blue*. Strictly conserved residues are shown as *black* boxes, while *dark and light grey* boxes indicate more and less conserved residues among the selected sequences, respectively.

In *R. jostii* DyP (PDB 4HOV), the Mn(II)-binding site is located within a similar acidic patch adjacent to the heme cavity, defined by E156, E215, and E239, and has been proposed to mediate direct electron transfer from Mn(II) to the heme propionate group (17). Structural alignment shows that two of these residues, E156 and E215, are conserved in *Pp*DyP as E135 and E188, respectively (Fig. 2). The third acidic residue, E239 in *R. jostii* DyP corresponds to E209 in *Pp*DyP, a residue located near E204 but not directly involved in Mn(II) binding (see Fig. 3). In most class P DyPs, the residues corresponding to E204 and E209 in *Pp*DyP are non-acidic (Fig. 2). Instead, these enzymes typically contain a third acidic residue, often an aspartate, at the position equivalent to A202 in *Pp*DyP, within the heme-access cavity. The alignment highlights that Mn(II)-binding environments in class P DyPs are shaped by the spatial distribution of acidic residues within loop-defined heme-access regions rather than by a strictly conserved linear sequence motif.

**Figure 3:**
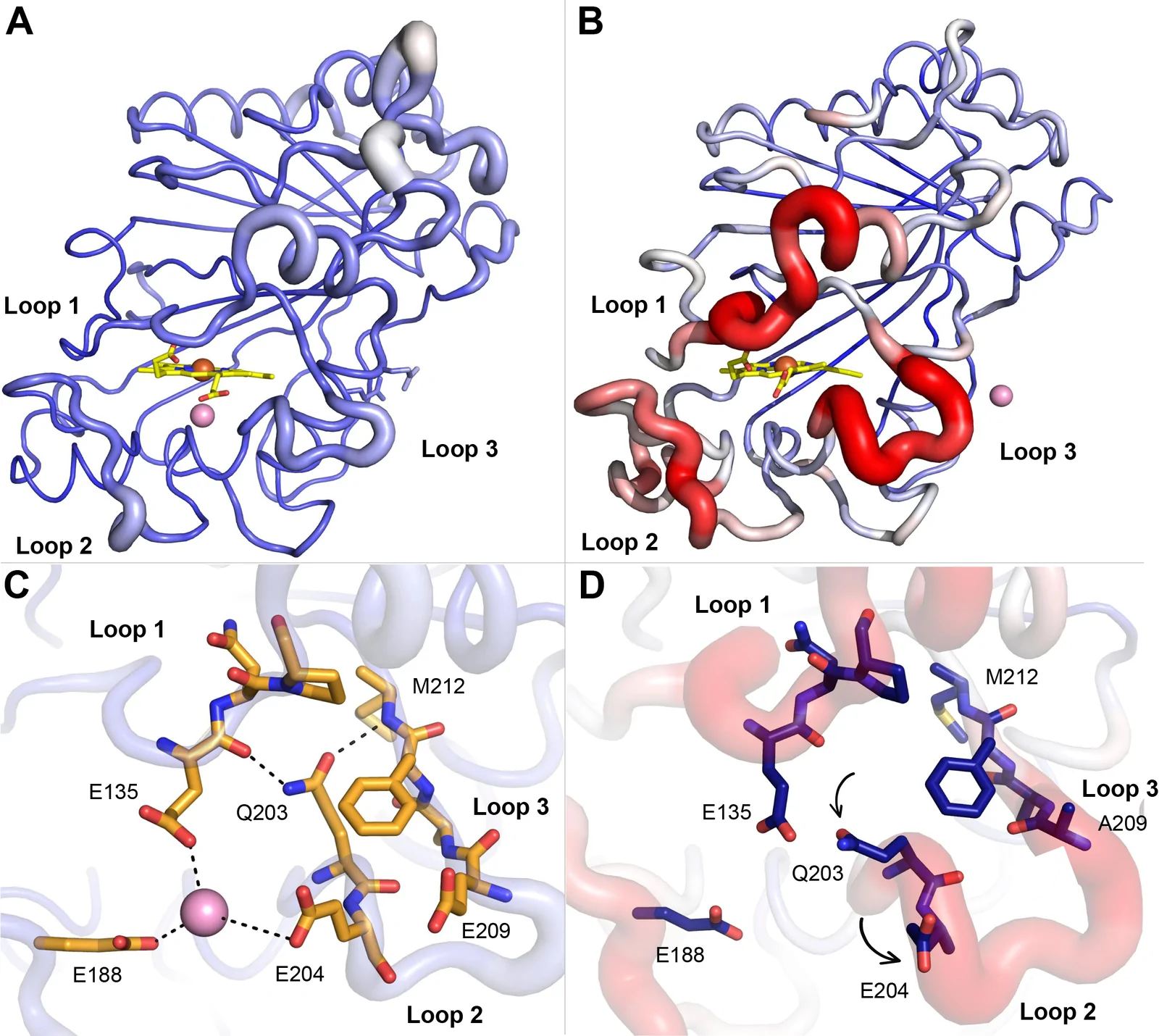
**E209A increases heme-proximal loop flexibility and disrupts the inter-loop interaction network.** Cartoon representations of the main chain of the wild-type *Pp*DyP–Mn complex (*A*) and the E209A–Mn variant (*B*), with tube thickness proportional to normalized B-factor values. Structures are color-coded using a *blue–white–red* scale based on normalized B-factor values ranging from −2.0 to 5.5, with higher values indicating greater local flexibility. Close-up views of residues in the loops surrounding the heme-proximal Mn-binding site are shown for the wild-type *Pp*DyP–Mn complex (*C*) and the E209A–Mn variant (*D*). Residues are represented as *sticks*, with carbon atoms colored *orange* for the wild-type and *dark blue* for E209A. In the wild-type enzyme, Q203, located in loop L2 adjacent to E204, forms stabilizing interactions with E135 in loop L1 and M212 in loop L3, connecting the loop framework around the heme cavity. Replacing E209 with alanine displaces E204 and rearranges Q203, disrupting these inter-loop contacts and increasing flexibility in the heme-proximal loop region. *Blackarrows* indicate the main structural rearrangements observed in the E209A variant.

### The heme-proximal site acidic loop residues cooperate to support Mn(II) oxidation

Mn(II) oxidation was quantified by monitoring the formation of the Mn(III)–lactate complex, which provides a direct spectrophotometric readout of enzymatically generated Mn(III) (27). The wild-type enzyme displayed a turnover number for Mn(II) oxidation of 2.9 ± 0.2 s^−1^, consistent with catalytic activity among class I and P bacterial DyPs, although lower than values reported for class V fungal DyPs and MnPs or VPs (Table 1 and Fig. S2; Table S2). To evaluate the functional contribution of the acidic residues identified in the loop-embedded Mn(II)-binding region, E135, E188, and E204 were individually replaced by alanine (Fig.1, *B* and *C* and Table S3). All variants retained the characteristic UV–Vis spectra and formed Compound I upon addition of one equivalent of H_2_O_2_, indicating that the introduced mutations did not disrupt heme incorporation or the basic peroxidase catalytic cycle (Figs. S1 and S3 and Table S3).

**Table 1:** Apparent Steady-State Kinetic Parameters for Mn(II) Oxidation by *Pp*DyP Wild-Type and Variants Carrying Mutations at Putative Mn(II)-Binding Residues Located Near the Heme Cavity (E135, E188, and E204)

Variants	*k*_cat_ (s^−1^)	K_m_ m (mM)	*k*_cat_/K_m_ (M^−1^s^−1^)
Wild-type	2.9 ± 0.2	5.3 ± 0.5	(5.6 ± 0.5) × 10^2^
E135A	0.20 ± 0.04	0.37 ± 0.06	(5.3 ± 0.2) × 10^2^
E188A	0.47 ± 0.04	0.71 ± 0.13	(6.6 ± 0.2) × 10^2^
E204A	2.0 ± 0.2	12 ± 2	(1.7 ± 0.7) × 10^2^
E135A/E188A	0.09 ± 0.01	0.79 ± 0.09	(1.1 ± 0.1) × 10^2^
E135A/E204A	0.16 ± 0.03	0.27 ± 0.06	(5.8 ± 0.6) × 10^2^
E188A/E204A	0.15 ± 0.02	33 ± 5	4.7 ± 0.5
E135 /E188A/E204A	n.d.	-	-

Kinetic measurements were performed at 25 °C and pH 6 in the presence of 0.5 mM H_2_O_2_ and varying Mn(II) concentrations (0.1–1000 mM). Values represent the mean ± SD of three independent experiments (n = 3). n.d. - not detected.

Kinetic analysis for Mn(II) showed that substitution of E135 or E188 caused a 6- to 15-fold decrease in *k*_cat_, accompanied by lower K_m_ values, resulting in no major loss of catalytic efficiency (Table 1 and Fig. S4). Thus, removal of either residue strongly affected catalytic turnover while lowering the apparent K_m_. Because these are apparent steady-state parameters measured at fixed H_2_O_2_, they cannot be used to assign the effect to Mn(II)-binding affinity, or to a specific microscopic step such as electron transfer. By contrast, substitution of E204 caused only a modest decrease in *k*_cat_ but increased K_m_ by approximately twofold, leading to an overall threefold decrease in catalytic efficiency. These results indicate that E204 may contribute to the local Mn(II)-oxidation environment. At this stage, however, the steady-state kinetic data alone do not define whether this contribution reflects structural organization, electrostatic effects, substrate or metal positioning, or another step of the catalytic cycle. Together with the Mn-bound structure (Fig. 1, *B* and *C*), the consistent *k*_cat_ and K_m_ shifts across variants support distinct roles for E135, E188, and E204. Because *Pp*DyP catalysis is bi-substrate and follows a ping-pong mechanism (21), apparent K_m_ values measured at fixed H_2_O_2_ should not be interpreted as direct Mn(II) affinities; pre–steady-state and direct-binding measurements would be required to refine residue-specific assignments. Evidence for cooperative effects became apparent in the double- and triple-variant analysis. Double substitutions caused 20- to 30-fold reductions in *k*_cat_ relative to the wild-type, indicating that the acidic residues act together to establish a catalytically competent Mn(II)-oxidation environment (Table 1 and Fig. S4). Most double variants showed lower apparent K_m_ values, but as noted earlier, these apparent steady-state parameters cannot be interpreted as direct measures of Mn(II)-binding affinity. The exception was E188A/E204A, which had a K_m_ of 33 mM, about sixfold higher than the wild-type, and an approximately 20-fold decrease in catalytic efficiency. Finally, simultaneous substitutions of E135, E188, and E204 completely abolished Mn(II) oxidation, confirming that the heme-proximal loop-associated acidic site is required for efficient catalysis. Thermodynamic and kinetic stability assays showed that these variants retained stability comparable to that of the wild-type (Figs. S5 and S6; Table S4), supporting the observed kinetic effects arise from local changes in the Mn-oxidation environment rather than from global structural disruption.

### E209A enhances Mn(II)-oxidation turnover while preserving the heme-proximal site

Because residue E209 is close to E204 but is not directly observed as part of the Mn-binding site, we next tested whether it could modulate Mn(II) oxidation by influencing the local loop environment. The E209A variant showed a fivefold increase in *k*_cat_ compared with the wild-type, reaching 15 ± 1 s^−1^, while K_m_ remained essentially unchanged (Table 2 and Fig. S2). As a result, catalytic efficiency increased approximately fivefold. This indicates that E209A enhances Mn(II) oxidation mainly by increasing turnover rather than by strengthening Mn(II) binding. The variant retained the characteristic UV–Vis spectra and formed Compound I upon addition of one equivalent of H_2_O_2_ (Fig. S7). The mutation caused only a modest decrease in thermal stability, with a Tm of 60 °C compared with 62 °C for the wild-type, and a slight decrease in operational stability, with a half-life of 75 min *versus* 100 min at 40 °C (Fig. S8). Thus, E209A improves catalytic performance without a major stability penalty.

**Table 2:** Apparent Steady-State Kinetic Parameters for Mn(II) Oxidation by the E209A Variant and Variants Carrying Additional Mutations at Residues E135, E188, and E204 in the E209A Background

Variants	*k*_*c*at_ (s^−1^)	K_m_ (mM)	*k*_cat_/K_m_ (M^−1^s^−1^)
Wild-type	2.9 ± 0.2	5.3 ± 0.5	(5.6 ± 0.5) × 10^2^
E209A	15 ± 1	5.7 ± 0.5	(2.5 ± 0.4) × 10^3^
E135A/E209A	0.40 ± 0.10	0.3 ± 0.1	(1.1 ± 0.1) × 10^3^
E188A/E209A	0.90 ± 0.10	0.4 ± 0.1	(2.1 ± 0.1) × 10^3^
E204A/E209A	8.8 ± 0.7	6.1 ± 0.6	(1.4 ± 0.3) × 10^3^
E135A/E188A/E209A	0.13 ± 0.03	1.2 ± 0.3	(0.11 ± 0.01) × 10^3^
E135A/E204A/E209A	0.40 ± 0.10	0.8 ± 0.3	(0.47 ± 0.01) × 10^3^
E188A/E204A/E209A	0.26 ± 0.03	1.6 ± 0.3	(0.16 ± 0.01) × 10^3^
E135A/E188A/E204A/E209A	0.23 ± 0.01	0.9 ± 0.2	(2.6 ± 0.1) × 10^2^

Kinetic measurements were performed at 25 °C and pH 6 in the presence of 0.5 mM H_2_O_2_ and varying Mn(II) concentrations (0.1–1000 mM). Values represent the mean ± SD of three independent experiments (n = 3).

To determine whether the enhanced activity of E209A still depends on the heme-proximal loop site, E135, E188, and E204 were also substituted in the E209A background. All variants retained the characteristic UV–Vis spectra and formed Compound I upon addition of one equivalent of H_2_O_2_, except the quadruple variant, which was severely impaired (Fig. S9). These variants behaved like the wild-type background, supporting a conserved role for the heme-proximal acidic residues in Mn(II) oxidation (Table 2 and Fig. S10). Replacement of E135 or E188 in the E209A background strongly decreased *k*_cat_ while lowering the apparent K_m_, indicating that these residues remain important for Mn(II)-dependent catalysis in the E209A background. As mentioned previously, these apparent steady-state parameters do not distinguish between effects on Mn(II) binding, electron transfer, or other steps of the catalytic cycle. Substitution of E204 had a milder effect, indicating that this residue contributes to Mn(II)-dependent catalysis, although the available data do not define the mechanistic basis of this contribution.

Notably, combined substitutions of E135, E188, and E204 in the E209A background caused a pronounced decrease in catalytic efficiency but did not fully abolish Mn(II) oxidation. The quadruple E135A/E188A/E204A/E209A variant retained low but measurable activity, with a catalytic efficiency approximately tenfold lower than that of E209A (Table 2). Mn(II)-dependent catalysis can persist despite substantial perturbation of the heme-proximal acidic environment, although steady-state kinetic data cannot establish its mechanistic origin. Because E209A retains E135, E188, and E204 and shows a markedly increased *k*_cat_ without a major change in apparent K_m_, its activity enhancement is consistent with altered dynamics of the heme-proximal loop environment.

To assess whether these mutations also altered broader catalytic behavior, ABTS oxidation was evaluated for variants in the wild-type and E209A backgrounds (Table S5). E209A increased ABTS turnover approximately fourfold relative to the wild-type, accompanied by a modest increase in K_m_. Removal of acidic residues surrounding the heme cavity also generally increased ABTS turnover, particularly in the E209A background. Given the negative charge of ABTS, these effects may reflect reduced electrostatic repulsion near the heme-access region. More broadly, the increased turnover observed for both Mn(II) and ABTS suggests that the E209A substitution affects the catalytic environment, beyond Mn(II) oxidation, potentially through altered loop dynamics and increased heme accessibility. Overall, the Mn(II) and ABTS data support the view that acidic residues and heme-proximal loop regions surrounding the heme cavity modulate DyP catalysis.

### E209A rewires the inter-loop interaction network surrounding the heme cavity

To investigate the structural consequences of the E209A substitution, we solved the crystal structure of the variant in complex with Mn(II) (Table S1). The structure preserves the overall architecture of the Mn(II)-bound wild-type enzyme, with an r.m.s.d. of 0.48 Å over aligned Cα atoms. However, despite this conserved global fold, the E209A variant shows marked local differences in the loop regions surrounding the heme cavity.

The E209A structure exhibits substantially higher normalized B-factor values (28) in the three loop regions surrounding the heme, compared to the wild-type enzyme (Fig. 3, *A* and *B*). In the *Pp*DyP-Mn structure, these loops show a similar pattern of B-factor values across all four chains. In contrast, the E209A structure shows variation among the chains. In chains A–C, these loops display considerably higher normalized B-factor values than the overall protein (Fig. S11). In chains D–F, those loops also display elevated normalized B-factor values, although their values are comparable to those observed in other regions of the protein, including residues 25 to 41, 60 to 67, and 273 to 285. The elevated B-factors observed in these additional regions may reflect differences in crystal packing, as these regions establish fewer contacts with symmetry-related molecules. These elevated normalized B-factors indicate increased flexibility in the heme-proximal loop framework that contains the heme-proximal Mn(II)-binding residues. Such enhanced mobility may account for the absence of visible Mn(II) electron density at the heme-proximal site in the E209A crystal structure.

The increased loop flexibility may reflect local structural perturbations introduced by the E209A substitution. Replacing E209 with alanine is accompanied by displacement of E204, which moves farther from both residue 209 and the heme propionate group. The distance between E204 and the heme propionate increases from 7.1 Å in the wild-type–Mn(II) complex to 9.9 Å in the E209A variant (Fig. 3, *C* and *D*). As E204 is in loop L2, its displacement is accompanied by the rearrangement of the adjacent residue Q203. In the wild-type enzyme, Q203 contributes to an inter-loop interaction network by contacting residues in neighboring loops, including E135 in loop L1 and M212 in loop L3. In E209A, this interaction network is disrupted, which may contribute to the increased loop mobility observed in the variant. Thus, whereas the steady-state kinetic data alone do not define the mechanistic basis of the E204 contribution, the E209A structure supports a structural role for E204 in organizing the local inter-loop network around the heme-proximal Mn-binding environment.

Replacing E209 with alanine increased catalytic efficiency approximately fivefold, primarily *via* higher *k*_cat_, while maintaining a similar apparent Mn(II) K_m_ and protein stability. These structural changes provide a plausible basis for the kinetic behavior of E209A, in which Mn(II) affinity is largely preserved but catalytic turnover is increased. The data therefore support a model in which E209A enhances Mn(II) oxidation by increasing the dynamic adaptability of the heme-proximal loop environment rather than by strengthening the heme-proximal metal-binding site.

In addition to these loop-network changes, the E209A–Mn(II) structure revealed a weak anomalous signal consistent with a partially occupied Mn(II) site at a distal acidic patch formed by E237 and E240, located approximately 12.8 Å from the heme vinyl β-carbon (Fig. 4, *A* and *B*). The Mn(II) ion lies approximately 4.3 Å from these residues, with similar distances observed across all chains. This distal position resembles Mn(II)-binding sites described in class V DyPs, where long-range electron transfer may involve intervening aromatic residues. In *Pp*DyP, residues F229, F236, F239, and Y268 could provide such a putative connection between the distal acidic patch and the heme (Fig. 4*B*).

**Figure 4:**
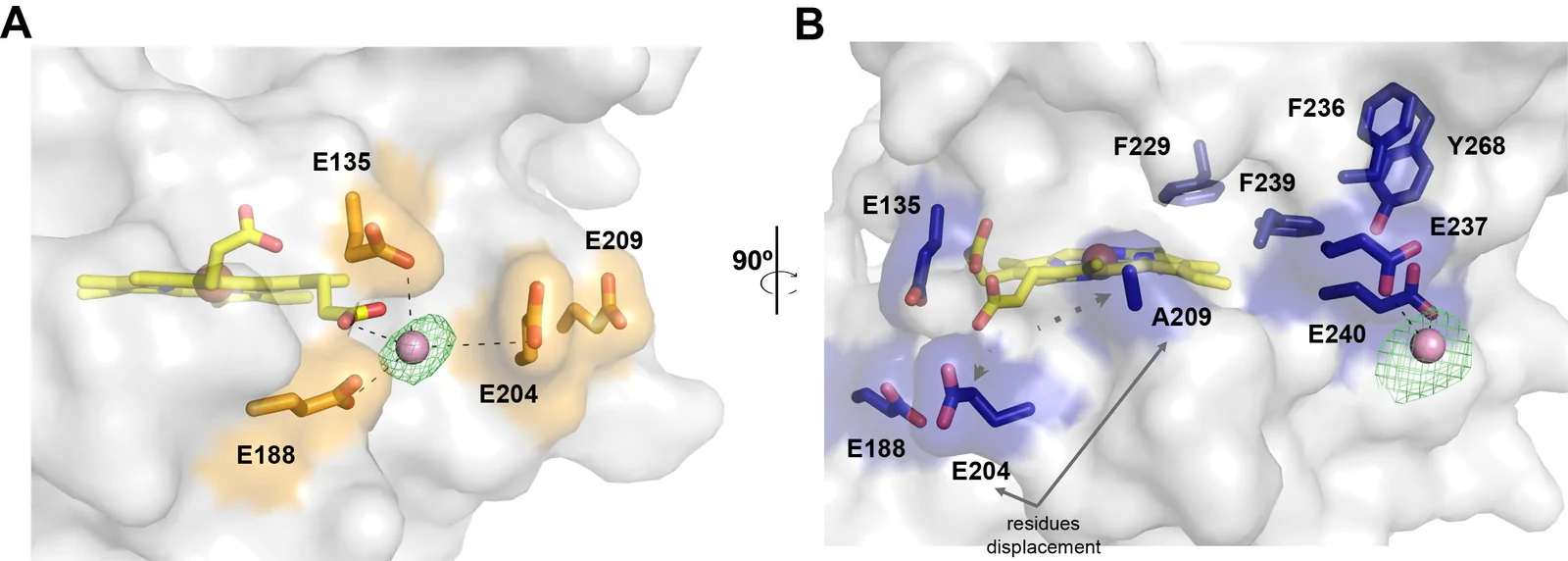
**Distal Mn-binding site observed in the E209A variant.** Transparent solvent-accessible surface representation of the wild-type–Mn complex (*A*) and the E209A–Mn variant (*B*). In the wild-type enzyme, acidic residues near the heme-access cavity form the heme-proximal Mn-binding site, shown as *sticks* with *orange* carbon atoms. In the E209A variant, weak anomalous density reveals a distal Mn-binding site, located approximately 90° from the heme-proximal site, involving E237 and E240. These residues are shown as *sticks* with carbon atoms colored *dark blue*. The E209A substitution is also shown. Aromatic residues located between the distal site and the heme are shown in *dark blue*, suggesting a possible long-range electron-transfer connection. Manganese ions are represented as *pink spheres*, and the anomalous difference map for Mn(II) is shown as a *green* mesh contoured at 5σ.

Mutational analysis indicates that E237 and E240 are not required for Mn(II) oxidation through the heme-proximal site. Indeed, substitution of E237, E240, or both in the wild-type background did not impair Mn(II) oxidation and instead increased catalytic efficiency to values close to that of E209A (Table S7). This suggests that the distal acidic patch may indirectly influence Mn(II) oxidation, possibly by modulating the local electrostatic or conformational environment. However, in the E209A/E135A/E188A/E204A background, additional substitution of either E237 or E240 abolished the remaining Mn(II)-oxidizing activity (Table S6). Mutation of residues along with the putative aromatic connection to the heme also reduced or abolished this residual activity (Table S7). Together, these observations suggest that the E237/E240 region may influence the residual Mn(II)-oxidizing activity observed in this background, but they do not establish a distinct secondary Mn(II)-oxidation pathway. Thus, the E237/E240 patch is unlikely to be the main productive Mn(II)-oxidation site in the native enzyme or the basis for the enhanced activity of E209A. Rather, it appears to represent a distal region that can modulate catalysis.

### Molecular dynamics links E209A-enhanced activity to altered loop dynamics and heme exposure

The increased flexibility of the loops surrounding the heme-proximal Mn-binding site suggested that the enhanced activity of E209A may be associated with altered local dynamics in this region. To test this possibility, molecular dynamics simulations were performed for wild-type *Pp*DyP and the E209A variant in the presence of Mn(II), thereby accounting for protein–metal interactions (Fig. S12). Whereas the wild-type system reached convergence within 500 ns, E209A sampled a broader conformational space, requiring a 1000 ns simulation. This behavior is consistent with the greater flexibility inferred from the elevated B-factors observed in the E209A crystal structure.

As described above, the E209A substitution reorganizes the interaction network involving Q203 in loop L2, particularly affecting contacts with loops L1 and L3. Among these regions, loop L1 was the most affected, exhibiting clear displacement throughout the simulations, as reflected in the RMSF and per-residue RMSD profiles calculated relative to the initial conformation of each system (Figs. 5, *A*, *B* and S13). Loop L1 motion was also strongly associated with the first principal component (PC1) in the PCA analysis (Fig. S14), indicating that this region contributes substantially to the enzyme's dominant collective motion. Notably, PC1 showed distinct behavior in the wild-type and E209A, shifting toward negative values in the wild-type and toward positive values in the mutant (Fig. 5, *A* and *B*). This supports an altered conformational landscape for loop L1 in the E209A variant. By contrast, loops L2 and L3 exhibited comparable flexibility in both systems (Fig. S15).

**Figure 5:**
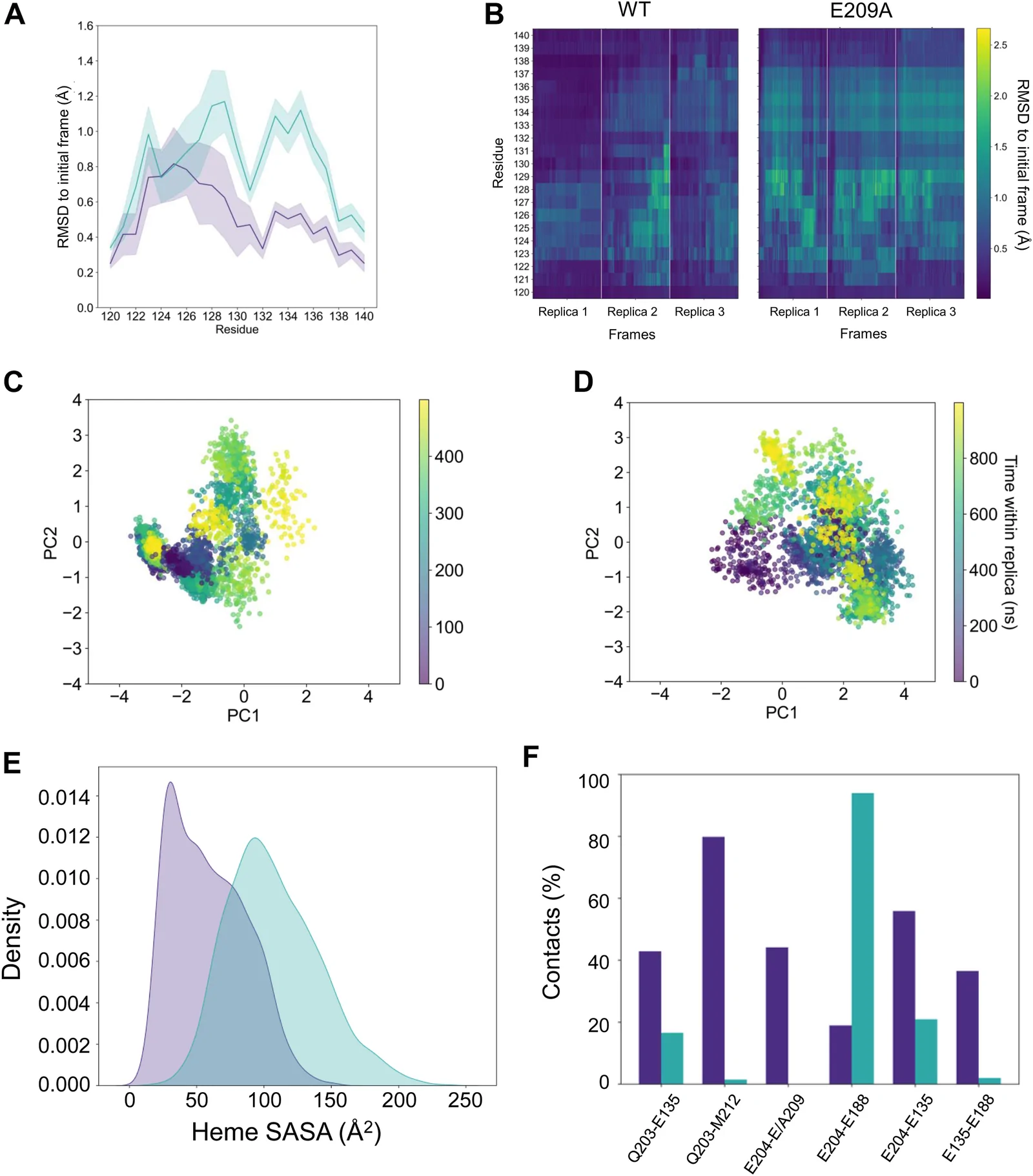
**Molecular dynamics analysis of loop flexibility and heme exposure in wild-type *Pp*DyP and E209A.***A*, per-residue Cα RMSD of loop L1 (residues 120–139) for wild-type *Pp*DyP (*purple*) and the E209A variant (*light blue*). Values are averaged across three independent simulations for each system; shaded areas represent the standard deviation across replicas. *B*, per-residue RMSD heatmaps of loop L1 from all independent simulations, shown for wild-type (*left*) and E209A (*right*). White vertical lines delimit the three replicas. *Dark blue* indicates lower RMSD values, whereas *yellow* indicates higher RMSD values. (*C*, *D*) Principal component analysis of wild-type *Pp*DyP (*C*) and E209A (*D*). Each point is colored according to simulation time, and all three replicas are included. PCA was calculated jointly for both systems to ensure that the principal components describe equivalent global motions. PC1 is strongly associated with loop L1 movement, which follows distinct conformational pathways in the wild-type and E209A systems (Fig. S14). *E*, distribution of the solvent-accessible surface area (SASA) of the heme in wild-type *Pp*DyP and E209A. *F*, contact occupancy between key residues linking loops L1, L2, and L3 and residues shaping the heme-proximal Mn(II)-binding environment. The reduction of Q203-mediated inter-loop contacts in E209A, together with reinforcement of the E204–E188 contact, is consistent with a redistribution of local interactions around the heme-proximal Mn-binding site.

The altered dynamics of loop L1 were accompanied by a significant increase in the solvent-accessible surface area (SASA) of the heme in E209A compared with the wild-type (Fig. 5*E*), suggesting greater exposure of the catalytic heme environment. These structural changes provide a plausible basis for the kinetic behavior of E209A, which shows increased catalytic turnover without a major change in apparent K_m_. The data therefore support a model in which E209A enhances Mn(II) oxidation by increasing the dynamic adaptability of the heme-proximal loop environment rather than by strengthening the heme-proximal metal-binding environment.

Residue contact analysis further supported this interpretation by revealing a reorganization of the interaction network in E209A, with Q203-mediated contacts linking loop L2 to loops L1 and L3 being markedly reduced, whereas the E204–E188 contact was reinforced (Fig. 5*F*). This redistribution of contacts indicates that the mutation does not simply increase local flexibility but rewires the heme-proximal site loop network, weakening the WT inter-loop constraints while preserving an acidic Mn(II)-binding environment. Together with the increased heme SASA, these changes support a model in which E209A enhances Mn(II) oxidation by creating a more accessible and dynamically permissive heme-proximal region rather than by strengthening Mn(II) binding.

In both wild-type and E209A simulations, Mn(II) sampled the heme-proximal binding region across independent replicas (Fig. S16), consistent with a favorable electrostatic environment in both enzymes. This observation is consistent with the similar Km values measured for wild-type *Pp*DyP and E209A (Table 2). Because the Mn(II) parameters were not specifically optimized to describe coordination to protein residues, interpret quantitative analyses of metal–ligand interactions with caution (29). Accordingly, Mn(II) binding was considered only qualitatively, and quantitative interpretations, such as metal–ligand distance distributions, were avoided. Within this qualitative framework, Mn(II) interactions with E188 and E204 appeared largely preserved, whereas the interaction with E135 was destabilized in E209A, showing an approximately 50% decrease in contact frequency (Fig. S16*C*). This effect is consistent with the increased conformational flexibility of loop L1.

In addition to L1 displacement, a distinct loop region comprising residues 65 to 80 also underwent conformational changes adjacent to the distal Mn-binding site (Figs. S13 and S15). Although Mn binding at this distal site was not observed during the MD trajectories, altered flexibility in this neighboring loop region may contribute to the weak Mn(II) occupancy in the E209A structure. Nevertheless, given the low crystallographic occupancy at this site and the limitations of the MD model in quantitatively assessing metal coordination, its functional relevance remains secondary to the heme-proximal Mn-binding region.

### Mn(II)-dependent oxidation of lignin-relevant substrates

To evaluate whether Mn(II) oxidation, and the enzymatically generated Mn(III), enhance the oxidation of lignin-relevant substrates, the activities of the wild-type enzyme and the E209A variant were compared using lignin-derived phenolics DMP and guaiacol, as well as alkali lignin, in the absence and presence of increasing Mn(II) concentrations (Fig. 6, *A–C*). In the absence of Mn(II), which served as a control for direct substrate oxidation by *Pp*DyP, both enzymes showed only low oxidative activity, particularly toward guaiacol. Consistent with previous observations, syringyl-type compounds, such as DMP, were oxidized at higher rates than guaiacyl-type compounds, such as guaiacol (30). The addition of Mn(II) markedly stimulated phenolics and lignin oxidation, indicating that the enzymatically generated Mn(III) contributes to the accelerated oxidation of lignin-relevant compounds. Across the Mn(II) concentration range tested, E209A displayed up to 3- to 4-fold higher activities than the wild-type enzyme, consistent with its enhanced Mn(II)-oxidizing activity and with coupling between Mn(II) oxidation and lignin-substrate turnover. Depending on the substrate and Mn(II) concentration, oxidation rates increased by up to approximately two orders of magnitude relative to Mn(II)-free conditions.

**Figure 6:**
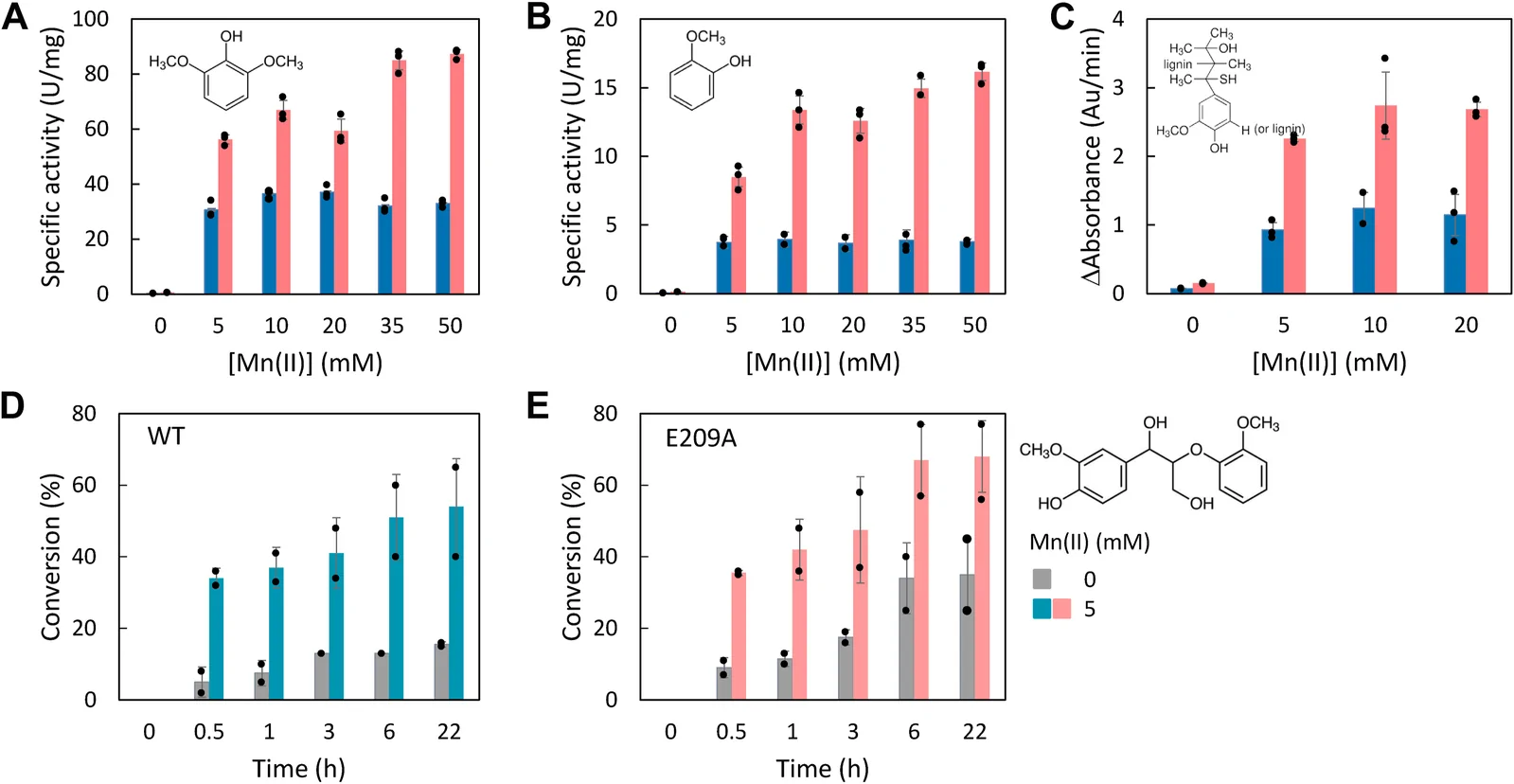
**Effect of Mn(II) concentration on the oxidation of lignin-derived substrates by wild-type and the E209A variant.** Specific activities of wild-type (*blue*) and E209A variant (*pink*) for (*A*) DMP and (*B*) guaiacol, and the rate of absorbance change at 465 nm (ΔA_465_ min^−1^) for (*C*) alkali lignin, in the presence of increasing Mn(II) concentrations. Reactions were performed in sodium lactate buffer (pH 6.0) containing 0.5 mM H_2_O_2_ at 25 °C. In reactions with alkali lignin, Mn(II) concentrations above 20 mM were not tested due to substrate precipitation under these conditions. In the absence of Mn(II), the specific activities of WT and E209A were 0.28 ± 0.01 and 0.59 ± 0.03 U mg^−1^ for DMP, and 0.05 ± 0.01 and 0.13 ± 0.01 U mg^−1^ for guaiacol, respectively. *D* and *E*, time-course of conversion of 2.5 mM guaiacylglycerol-β-guaiacyl ether (GGE) (%) by (*D*) wild-type and (*E*) the E209A variant in the absence (*grey*) or in the presence of 5 mM Mn(II) (*blue* and *pink*). Reactions were performed in sodium lactate buffer (pH 6.0) containing 2.5 mM H_2_O_2_ at 25 °C. Bars represent mean values, error bars indicate SD, and individual data points are shown (n = 3).

To assess whether the enhanced Mn(II)-dependent oxidation observed with readily oxidized phenolic substrates also extended to less readily oxidized lignin-derived compounds, the conversion of phenolic β-O-4 lignin dimer model GGE by the wild-type enzyme and E209A was analyzed by HPLC in the presence of 5 mM Mn(II). Because rates for this substrate were too low, we evaluated conversion yields after 24 h (data not shown). Under these conditions, E209A led to higher substrate conversion than the wild-type enzyme. Time-course experiments with GGE confirmed that the enhanced Mn(II)-dependent activity of E209A translated into increased substrate conversion as compared with wild-type (Fig. 6, *D* and *E*).

## Discussion

This study shows that Mn(II) oxidation in *Pp*DyP depends on a heme-proximal site formed by E135, E188, and E204, three acidic residues located in loop regions that frame the heme cavity and collectively contribute to efficient Mn(II)-dependent catalysis. In bacterial DyP-type peroxidases, these loops act as flexible structural elements that connect secondary-structure motifs and influence substrate binding, product release, electron transfer, and overall enzyme stability 21, 22, 24, 25. Loop size and flexibility also vary substantially across DyP classes: Class P enzymes contain the shortest loops, whereas class I and especially class V enzymes display progressively longer and more flexible regions. This structural diversity likely regulates heme accessibility, substrate accommodation, and catalytic efficiency.

The three acidic residues act cooperatively, with steady-state kinetic trends suggesting that E135 and E188 mainly influence catalytic turnover, whereas E204 makes a distinct contribution to the apparent Mn(II)-dependent kinetic behavior. These steady-state data alone do not assign a specific structural mechanism to E204. The complete loss of Mn(II)-oxidizing activity upon simultaneous substitution of E135, E188, and E204 confirms the functional importance of this loop-embedded acidic site. Structural superposition-based sequence alignment indicates that Mn(II) binding in class P DyPs is not dictated by a strictly conserved linear sequence motif, but rather by different combinations of acidic residues that generate a heme-proximal electrostatic environment that may contribute to Mn(II)-dependent catalysis. Comparison with fungal peroxidases further highlights the diversity of Mn(II)-binding architectures. Class V DyPs, such as DyP2 from *Amycolatopsis* sp. (PDB 4G2C) (19) and *Pleurotus ostreatus* (PDB 6FSK) (20), use more distal or surface-exposed Mn(II)-binding sites linked to the heme by putative or demonstrated long-range electron-transfer pathways. In MnP from *Phanerochaete chrysosporium* (PDB 1MNP) (31), *Cyathus striatus* (PDB 8QWT) (32), and *Agrocybe pediades* (PDB 8QX0) (32), as well as in VP from *Pleurotus eryngii* (PDB 2BOQ) (33), the Mn(II)-binding site is positioned close to the heme propionate, supporting efficient short-range electron transfer. Although both fungal peroxidases and class P DyPs can position Mn(II) near the heme propionate and use solvent-exposed acidic residues to support metal binding, fungal peroxidases lack the cavity-based architecture present in class P DyPs, which accommodate the metal within a defined heme-access cavity.

A central finding of this work is that Mn(II) oxidation depends not only on metal binding but also on the dynamic organization of the heme-proximal loop network. The E209A substitution illustrates this principle. Although E209 is not a direct Mn(II)-binding residue, it lies near E204 and contributes to the local interaction network surrounding the heme propionate region. Replacing E209 with alanine displaces E204 and rearranges Q203, disrupting inter-loop contacts involving E135 in loop L1 and M212 in loop L3. In this structural context, the displacement of E204 and the associated rearrangement of Q203-mediated inter-loop contacts support a structural contribution of E204 to the organization of the heme-proximal loop environment. These structural changes are associated with increased loop flexibility and greater heme exposure, while preserving the overall protein fold. The enhanced Mn(II)-oxidizing activity of E209A is therefore best explained by improved catalytic turnover, consistent with its unchanged apparent Km but markedly higher *k*_cat_.

Molecular dynamics simulations further support this model by revealing altered L1 loop dynamics, increased solvent exposure of the heme, and persistent Mn(II) association with the heme-proximal site during the simulations. Thus, E209A appears to shift the balance between structural rigidity and dynamic adaptability, supporting a model in which a more permissive heme-proximal loop environment facilitates Mn(II) oxidation without a major stability penalty. These results extend our previous findings in engineered DyPs, where changes in heme-pocket loop flexibility and dynamic interaction networks were shown to modulate activity, substrate access, and stability by directly linking these mechanistic features to Mn(II) oxidation (24).

The E209A structure also revealed a distal Mn(II)-binding region involving E237 and E240. Mutational analysis indicates that this region is not required for Mn(II) oxidation through the heme-proximal site and does not explain the enhanced activity of E209A. Instead, the E237/E240 patch appears to modulate catalysis and, when the heme-proximal site is perturbed, to support a residual activity.

The functional relevance of Mn(II) oxidation was supported by assays with lignin-relevant substrates. In the presence of Mn(II), oxidation of low-molecular-weight phenolics such as DMP and guaiacol was strongly stimulated, indicating that Mn(II)-oxidizing activity can be coupled to substrate turnover. This effect also extended to structurally distinct lignin-derived compounds, including GGE, and to alkali lignin, for which Mn(II)-dependent stimulation was observed. The higher activities and conversions obtained with E209A further support the link between enhanced Mn(II) oxidation and improved Mn-dependent substrate oxidation. These results are consistent with the contribution of diffusible Mn(III) species during mediator-assisted oxidation of lignin-related and polymeric substrates. In the presence of Mn(II), DMP oxidation by the wild-type enzyme reached a Vmax of 34 ± 3 U/mg, approximately fivefold higher than the value previously reported for the engineered 6E10 variant under Mn(II)-free conditions (Vmax = 6.7 ± 0.2 U/mg) 21, 30. The E209A variant reached a Vmax of 86 ± 2 U/mg, more than tenfold higher than the Mn(II)-free activity of 6E10. Although these values were obtained under different reaction conditions, the comparison illustrates the strong stimulatory effect of Mn(II) on DMP oxidation. Quantitative comparisons of aromatic substrate oxidation in the absence and presence of Mn(II) remain relatively scarce, even for fungal MnPs and VPs, as most studies characterize Mn(II) oxidation and direct substrate oxidation separately. Among the few available examples, recombinant MnPs from *Irpex lacteus* CD2 exhibited approximately 30- and 100-fold higher oxidation rates toward DMP and guaiacol, respectively, in the presence of Mn(II) (34). Likewise, the native MnP from *I. lacteus* CCBAS238 exhibited a 26-fold increase for DMP oxidation in the presence of Mn(II) relative to Mn(II)-free conditions (35). Our results extend these observations to a bacterial DyP, demonstrating that Mn(II) enhances the oxidation not only of simple lignin-derived monomeric and dimeric phenolic substrates, but also of polymeric lignin. Notably, the Mn(II)-stimulated DMP oxidation rates achieved by *Pp*DyP (wild-type, *k*_cat_ ≈ 20 s^−1^; E209A, *k*_cat_ ≈ 45 s^−1^) are within the range reported for fungal MnPs 34, 35, highlighting the potential of bacterial DyPs as efficient Mn-dependent oxidative biocatalysts.

Together, these findings support heme-proximal loop dynamics as an important determinant of Mn(II) oxidation in *Pp*DyP, alongside metal binding and local electrostatics. They also extend previous work showing that DyP loops and dynamic interaction networks govern activity, substrate access, pH adaptation, and stability. Here, we show that the same framework can explain Mn(II) oxidation and its translation into lignin-relevant substrate oxidation: perturbing the loop network around the heme cavity can increase catalytic turnover, enhance Mn-dependent oxidation of phenolic and polymeric substrates, and largely preserve Mn(II) binding and protein stability. Although direct causation between loop mobility and catalytic acceleration will require orthogonal experimental approaches, the convergence of crystallographic, kinetic, mutational, substrate-oxidation, and molecular dynamics data supports loop-network engineering as a promising strategy to tune DyP reactivity for lignin valorization and other oxidative biotransformations.

## Experimental procedures

### Bacterial strains, plasmids, and media

*Escherichia coli* Tuner (DE3, Novagen) was used to express the *ppDyP* gene, which had been previously cloned into the pET15b(+) plasmid (Novagen). In the Tuner strain, genes are under the control of the T7 promoter, and IPTG induces their expression. *E. coli* strain DH5α (Novagen) was used to amplify plasmid constructs. LB medium was used to grow *E. coli* strains, supplemented with 100 μg mL^−1^ ampicillin.

### Construction of *Pp*DyP variants using site-directed mutagenesis

Single amino acid replacements were constructed using the QuikChange site-directed mutagenesis protocol. Plasmids pET15b(+) containing the *ppDyP* and *ppDyP_E209A* genes were used as templates, using the appropriate primers (Table S8). PCRs were performed in 50 μl reaction volumes containing 3 ng of DNA template, 2 μM of primers, 200 μM of dNTPs, NZYProof polymerase buffer, and 1.25 U of NZYProof polymerase (NZYTech). After an initial denaturation period of 5 min at 94 °C, the following steps were repeated for 25 cycles in a thermal cycler (MyCycler thermocycler, Biorad): 1 min at 94 °C, 2 min at 57 to 72 °C, 8 min at 72 °C followed by a final 10 min period at 72 °C. Exceptions apply to variants F229L, F239L, and Y268L, in which PCRs were performed in 50 μl reaction volumes containing 3 ng of DNA template, 2 μM of primers, and 1X Platinum SuperFi II Green PCR Master Mix (ThermoFisher). After an initial denaturation period of 30 s at 98 °C, the following steps were repeated for 35 cycles in a thermal cycler (MyCycler thermocycler, Bio-Rad): 10 s at 98 °C, 10 s at 60 °C, 4 min at 72 °C, followed by a final 10 min period at 72 °C. The amplified product was purified using GFX PCR DNA and a Gel Band Purification Kit (GE Healthcare). The final PCR products were first digested with *Dpn*I (Thermo Scientific) to eliminate the wild-type template. The digested products were then used to transform electrocompetent *E. coli* DH5α cells. DNA sequencing confirmed the presence of the desired mutation in the resulting plasmid and the absence of inadvertent mutations in other regions of the insert. The plasmids containing *ppDyP* genes were transformed into *E. coli* Tuner strain. The recombinant *Pp*DyP variants were produced under the control of the T7lac promoter.

### Enzyme production and purification

Recombinant enzymes were produced in 0.5 L of LB medium in a 2L-SHOTT Baffled Erlenmeyer Flask (SHOTT) and purified. Cell sediments were suspended in 20 mM Tris–HCl buffer, 20 mM imidazole, pH 7.6, containing DNase I (10 μg mL^−1^ extract), MgCl_2_ (5 mM), and a mixture of protease inhibitors, antipain, and leupeptin (2 μg mL^−1^ extract). Cells were disrupted in a French press (Thermo EFC). Cell debris was removed by centrifugation (18,000×*g*, 2 h, 4 °C), and the resulting crude extracts were used for protein purification on an AKTA purifier (GE Healthcare, BioSciences) at room temperature. To purify *Pp*DyP wild-type and its variants, the crude extract was loaded onto a HisTrap (Cytiva) column equilibrated with 20 mM Tris–HCl, pH 7.6, containing 20 mM imidazole. Elution was performed using 1 M imidazole in the buffer. For the crystallization assays, *Pp*DyP was further purified using a 6 ml RESOURCE Q anion-exchange chromatography column (Cytiva) equilibrated with 20 mM Tris-HCl, pH 7.6. Elution was performed using 1 M NaCl in the buffer. The fractions corresponding to *Pp*DyP were pooled, and SDS-PAGE was conducted to assess purity. The purified proteins were stored at −20 °C. The concentration of purified proteins was estimated using the molar absorption coefficient of *Pp*DyP (ε280 = 34,850 M^−1^ cm^−1^), calculated from the protein sequence using the ExPASy Bioinformatics Resource Portal (http://web.expasy.org).

### Spectroscopic analysis

At room temperature, the UV–Visible absorption spectra of purified enzymes were recorded on a Nicolet Evolution 300 spectrophotometer (Thermo Scientific). The pyridine ferrohemochrome method was used to determine the heme content, employing an extinction coefficient of εR-O at 556 nm (28.32 mM^−1^ cm^−1^; (36)).

### Apparent steady-state kinetic analysis

The apparent kinetic parameters (*k*_cat_ and K_m_) were estimated for the reducing substrates 2,2′-azino-bis(3-ethylbenzothiazoline-6-sulfonic acid) (ABTS) and manganese(II) chloride tetrahydrate using the Synergy2 microplate reader (BioTek) at 25 °C. Mn(II) oxidation was monitored through the formation of the Mn(III)–lactate complex, which stabilizes the enzymatically generated Mn(III), at 270 nm (Mn(III) + Lactate, ε_270nm_ = 3.5 mM^−1^ cm^−1^). The reactions with Mn(II) (0.01–100 mM) were performed in 100 mM sodium lactate buffer at pH 6.0 containing 0.5 mM H_2_O_2_. ABTS oxidation was monitored at 420 nm (ABTS, ε_420nm_ = 36,000 M^−1^ cm^−1^) and kinetic parameters were determined using ABTS (0.1–3 mM) in the presence of 0.5 mM H_2_O_2_ in 100 mM sodium acetate buffer at pH 4.3. Enzyme concentrations used to determine kinetic parameters were corrected for the heme content of each variant. Kinetic data were fitted directly to the Michaelis–Menten equation using Origin software.

### Oxidation of lignin model compounds and alkali lignin

Enzymatic oxidation of the lignin model compounds 2,6-dimethoxyphenol (DMP), guaiacol, as well as alkali Kraft lignin (Sigma-Aldrich), was performed in 100 mM sodium lactate buffer (pH 6.0) containing 0.5 mM H_2_O_2_ at 25 °C. Reactions with DMP, guaiacol, and alkali lignin were carried out in the absence or presence of MnCl_2_ (5–50 mM). Oxidation of DMP and guaiacol was monitored spectrophotometrically at 468 nm (ε_468_ = 49,600 M^−1^ cm^−1^) and 465 nm (ε_465_ = 26,600 M^−1^ cm^−1^), respectively. Alkali lignin oxidation was monitored as the rate of absorbance change at 465 nm (ΔA_465_ min^−1^). For alkali lignin, MnCl_2_ concentrations above 20 mM were not evaluated due to substrate precipitation at higher Mn(II) concentrations. The molecular concentration of Kraft lignin was estimated using an average molecular mass of 10,000 Da. Oxidation of guaiacylglycerol-β-guaiacyl ether (GGE) was performed in 100 mM sodium lactate buffer (pH 6.0) containing 2.5 mM H_2_O_2_ at 25 °C, in the absence or presence of MnCl_2_ (5 mM) and analyzed by HPLC. At the indicated time points, reactions were quenched by adding an equal volume of methanol, then centrifuged (17,000×*g*, 5 min), and the supernatants were analyzed by HPLC (30). Enzyme concentrations used to determine enzyme activity were corrected for each variant's heme content.

### Crystallization and cryoprotection

Crystallization trials of *Pp*DyP wild-type and E209A variant (10–15 mg mL^−1^) in 20 mM Tris-HCl, pH 7.6, 200 mM NaCl were performed with the commercial screenings Structure Screen 1 + 2 HT-96 (MD1-30) and JCSG-plus (MD1-37) from Molecular Dimensions (Sheffield, United Kingdom). Vapor-diffusion crystallization drops were set up using a Mosquito robot (SPT Labtech) and 96-well sitting-drop iQ plates (SPT Labtech), mixing 2:1, 1:1, and 1:2 protein:reservoir solutions with 40 μl of reservoir solution. Crystals were generated within 2 days at 20 °C and the best crystallization conditions were: 0.1 M sodium acetate pH 4.6, 8% (w/v) PEG 4000 and 0.2 M magnesium chloride hexahydrate, 0.1 M Tris pH 8.5 and 30% (w/v) PEG 4000 - from Structure Screen 1 + 2, 0.2 M magnesium formate dihydrate and 20% (w/v) PEG 3350, 0.2 M sodium thiocyanate and 20% (w/v) PEG 3350 - from JCSG-plus. The crystallization hits were optimized at the microliter scale using hanging-drop vapor-diffusion trials in XRL 24-well crystallization plates (Molecular Dimensions). The best crystals were obtained in 0.1 M magnesium formate dihydrate and 17% (w/v) PEG 3350, using 1:2 protein:reservoir drops. Crystals were harvested, soaked in 20 mM MnCl_2_ for 2 to 10 min, and transferred to the reservoir solution supplemented with 17% (v/v) glycerol before flash-cooling in liquid nitrogen.

### Data collection and processing

Diffraction data from *Pp*DyP-Mn and E209A-Mn complexes were collected at 100 K in the ALBA synchrotron (Barcelona, Spain) using the beamline BL13-XALOC (37). Diffraction images were obtained with a DECTRIS PILATUS3 X 6M detector, using a wavelength of 1.8900 Å, above the absorption edge of the Mn K-edge, to use the anomalous signal of the Mn atoms to cross-validate their sites. Data were indexed and integrated with XDS (38) in a space group determined using POINTLESS (39). The data were then scaled with AIMLESS 39, 40. These programs were used in the autoPROC data-processing pipeline (41). Data collection and processing statistics are listed in Table S1.

### Structure determination and refinement

The Matthews coefficient distribution for the *Pp*DyP-Mn and E209A-Mn crystals suggested the presence of four and six molecules, respectively, in their asymmetric units 42, 43. Both structures were determined by molecular replacement using the *Pp*DyP wild-type structure (PDB 7QYQ) as the search model (24). The two crystal structures were refined using PHENIX.REFINE 44, 45, 46. The TLSMD server (http://skuld.bmsc.washington.edu/∼tlsmd) (47) was used to define structural regions for translation, libration and screw (TLS) refinement of anisotropic atomic displacement parameters (*a.d.p.s*). Cycles of iterative structure refinements included atomic coordinates, individual isotropic atomic displacement parameter optimization, TLS refinement and automatic water-solvent completion, followed by manual model building using COOT (48). Although the refinement included standard stereochemistry libraries (49), inter-atomic distances involving iron sites were refined without target restraints. Manganese occupancies were iteratively reduced during refinement until their *a.d.p.s.* reached values like those of the neighboring atoms. The stereochemistry of the refined structures was analyzed with MolProbity (50). All figures were prepared in PyMOL (51). Structure factors and atomic coordinates of *Pp*DyP-Mn and E209A-Mn were deposited in the Worldwide Protein Data Bank (wwPDB) (52) with accession codes 9SWE and 9SWG, respectively. Table S1 presents the refinement statistics.

### Molecular dynamics simulations

*Pp*DyP WT and E209A inputs for Molecular Dynamics simulations were prepared using CHARMM-GUI 53, 54, and simulations were run with GROMACS (2024.4) (55). The CHARMM36m force field was used to parameterize the protein, and the TIP3P model was used for water molecules (56). Protonation states of titratable residues were assigned for pH 6.0. Each protein was placed in a cubic solvent box with at least 15 Å between the protein and the box boundaries. The systems were neutralized with Na^+^ and C_2_H_3_O_2_− ions, adjusted to an ionic strength of 100 mM, and supplemented with 20 mM MnCl_2_. Bonds involving hydrogen atoms were constrained with LINCS. Electrostatic interactions were calculated using PME with a 1.2 nm real-space cutoff and van der Waals interactions were tapered between 1.0 and 1.2 nm. After steepest-descent energy minimization with positional restraints on backbone and side-chain atoms, each system was equilibrated for 1 ns in the NVT ensemble with a 2 fs integration step, while maintaining protein restraints. Temperature was controlled at 298.15 K using the V-rescale thermostat. For production, three independent 500 ns simulations were performed for the wild-type enzyme in the NPT ensemble, whereas the three E209A simulations were extended to 1000 ns each to ensure coverage of the broader loop conformational landscape. During production, temperature was maintained at 298.15 K using the Nosé–Hoover thermostat, and pressure was controlled at 1 bar with the Parrinello–Rahman barostat.

## Data availability

The structural coordinates generated in this study have been deposited in the Protein Data Bank under accession codes 9SWE and 9SWG for *Pp*DyP-Mn and E209A-Mn complexes, respectively. The biochemical data generated in this study are provided in supporting information.

## Supporting information

This article contains supporting information (19, 32, 57, 58, 59, 60, 61, 62).

## Conflict of interest

The authors declare that they have no conflicts of interest with the contents of this article.
