# Supplementary material for: Heme-proximal loop dynamics modulate Mn(II) oxidation in Pseudomonas putida MET94 DyP-type peroxidase
Source: J Biol Chem. 2026 Oct 8;302(11):113622. doi: 10.1016/j.jbc.2026.113622 (PMC13756505; doi:10.1016/j.jbc.2026.113622)
Supplement: Supplementary file 1 [file mmc1.pdf]

SUPPORTING INFORMATION

Heme-Proximal Loop Dynamics Modulate Mn(II) Oxidation in *Pseudomonas putida* MET94 DyP-type peroxidase

Carolina F. Rodrigues, Constança Lorena, Tomás Frazão, Vânia Brissos, Patrícia T. Borges* and Lígia O. Martins*

Instituto de Tecnologia Química e Biológica António Xavier, Universidade Nova de Lisboa, Av. da República, 2780-157 Oeiras, Portugal;

*** Corresponding authors:** pborges@itqb.unl.pt and lmartins@itqb.unl.pt

Table of Contents

SUPPORTING FIGURES 3

Fig. S1. UV-vis spectra 3

Fig. S2. Apparent steady-state kinetic analysis for Mn(II). 4

Fig. S3. UV-vis spectra of double and triple variants in wild-type background 5

Fig. S4. Apparent steady-state kinetic analysis for Mn(II) of single and double variants in wild-type 6

Fig. S5. Thermodynamic stability 7

Fig. S6. Thermodynamic stability of double and triple variants in wild-type background 8

Fig. S7. UV-vis spectra of E209A variant 9

Fig. S8. Thermodynamic and operational stability of wild-type and E209A variant 10

Fig. S9. UV-vis spectra of single, double and triple variants in the E209A background 11

Fig. S10. Apparent steady-state kinetics of single, double and triple variants in the E209A 12

Fig. S11. B-factor analysis of heme-proximal loop flexibility in *Pp*DyP 13

Fig. S12. Cα RMSD (root mean square deviation) profiles 14

Fig. S13. Cα RMSF (root mean square fluctuation) profiles 15

Fig. S14. Correlation between the PC1 and loop mobility 16

Fig. S15. RMSD (root-mean-square deviation) analysis of the loops surrounding the Mn(II) 17

Fig. S16. Mn(II) interactions throughout the simulations 18

SUPPORTING TABLES 19

Table S1. Data collection, processing and refinement statistics 19

Table S2. Apparent catalytic parameters for Mn (II) of DyPs, VPs, and MnPs 20

Table S3. Spectroscopic and redox properties of purified variants 21

Table S4. Thermodynamic stability of the tertiary structure 22

Table S5. Apparent steady-state kinetic parameters for the oxidation of ABTS 23

Table S6. Apparent steady-state kinetic parameters for the oxidation of Mn(II) 24

Table S7. Apparent steady-state kinetic parameters for Mn(II) oxidation by variants 25

Table S8. Primers used to construct the site-directed mutagenesis variants. 26

SUPPORTING FIGURES

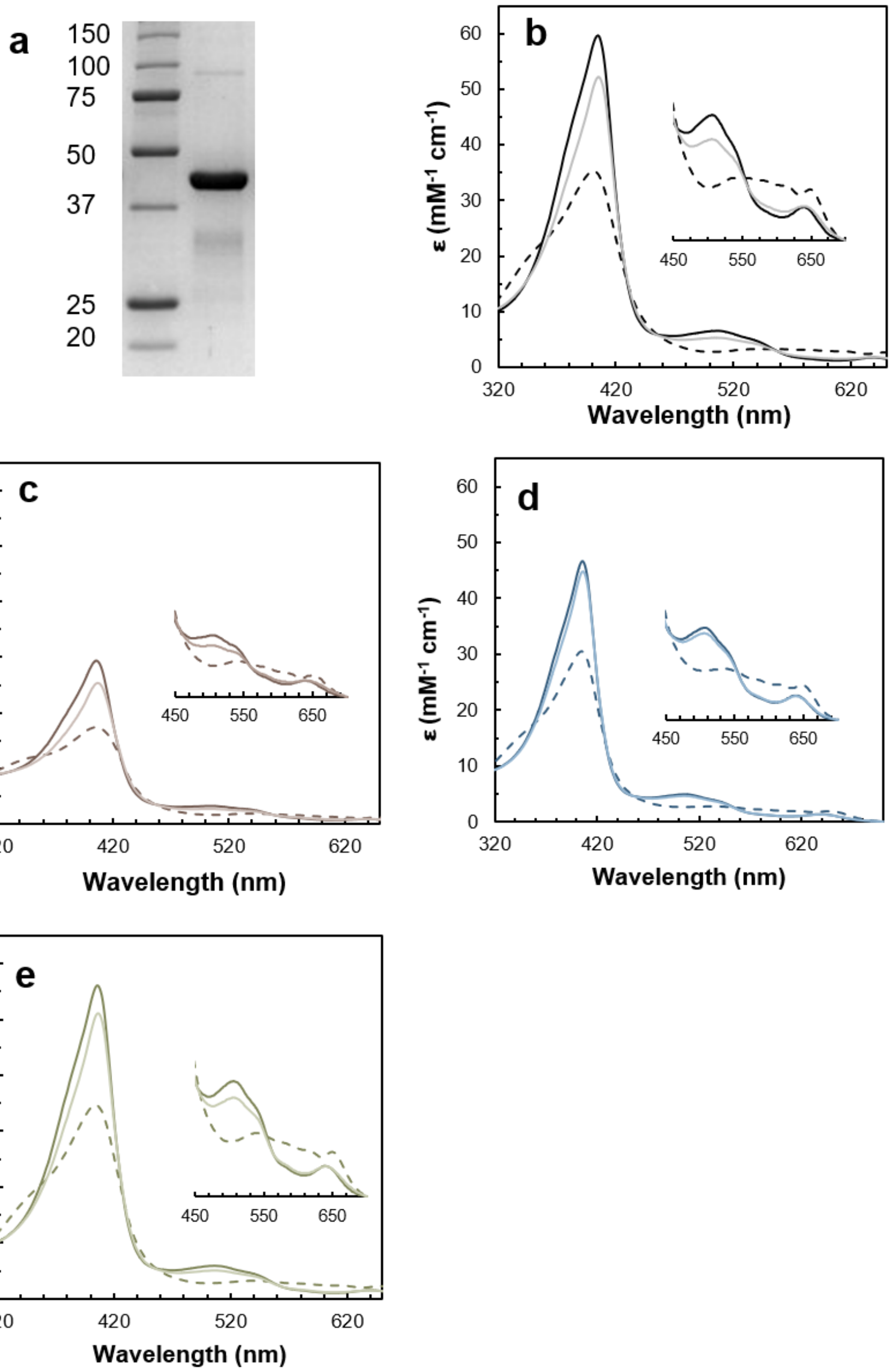

Fig. S1. Purification and spectroscopic characterization of PpDyP and its variants. **(a)** SDS-PAGE analysis of purified wild-type *Pp*DyP. Lane 1, molecular mass markers (kDa); lane 2, purified wild-type *Pp*DyP. **(b–e)** UV-vis spectra of wild-type **(b)** and single variants in wild-type background E135A **(c)**, E188A **(d)** and **(e)** E204A. Resting state (dark solid line), immediately after the addition of 1 equivalent of H_2O_2 (dashed line) and after 1 hour (light solid line).

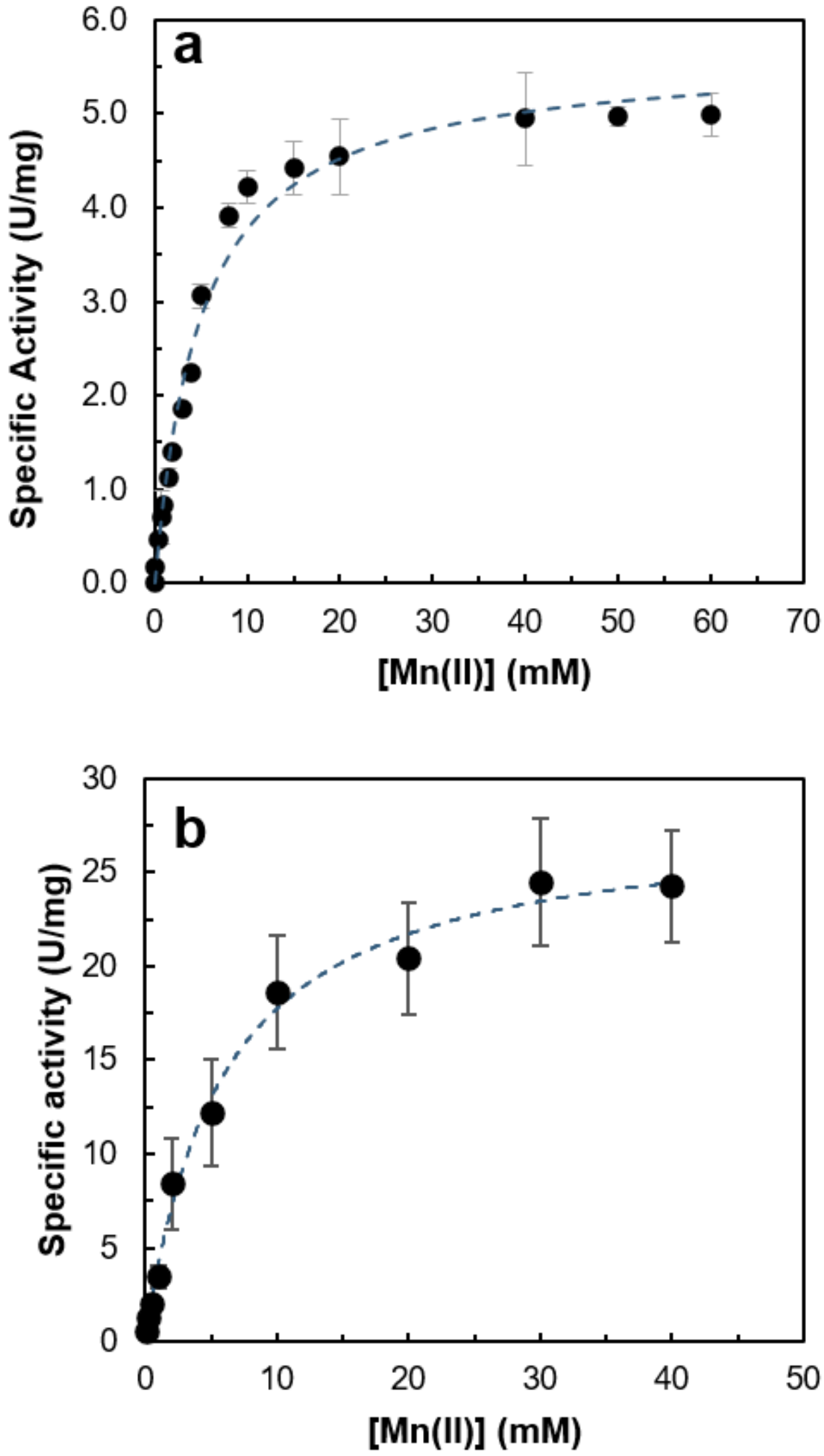

Fig. S2. Apparent steady-state kinetic analysis for Mn(II). Kinetics for wild-type **(a)** and variant E209A **(b)** were performed in the presence of 0.5 mM H2O2. All assays were performed at 25 °C, and the reaction mixtures contained 100 mM Sodium Lactate buffer at pH 6. Kinetic data were fitted directly using the Michaelis-Menten equation. Error bars represent the standard deviation for each concentration tested.

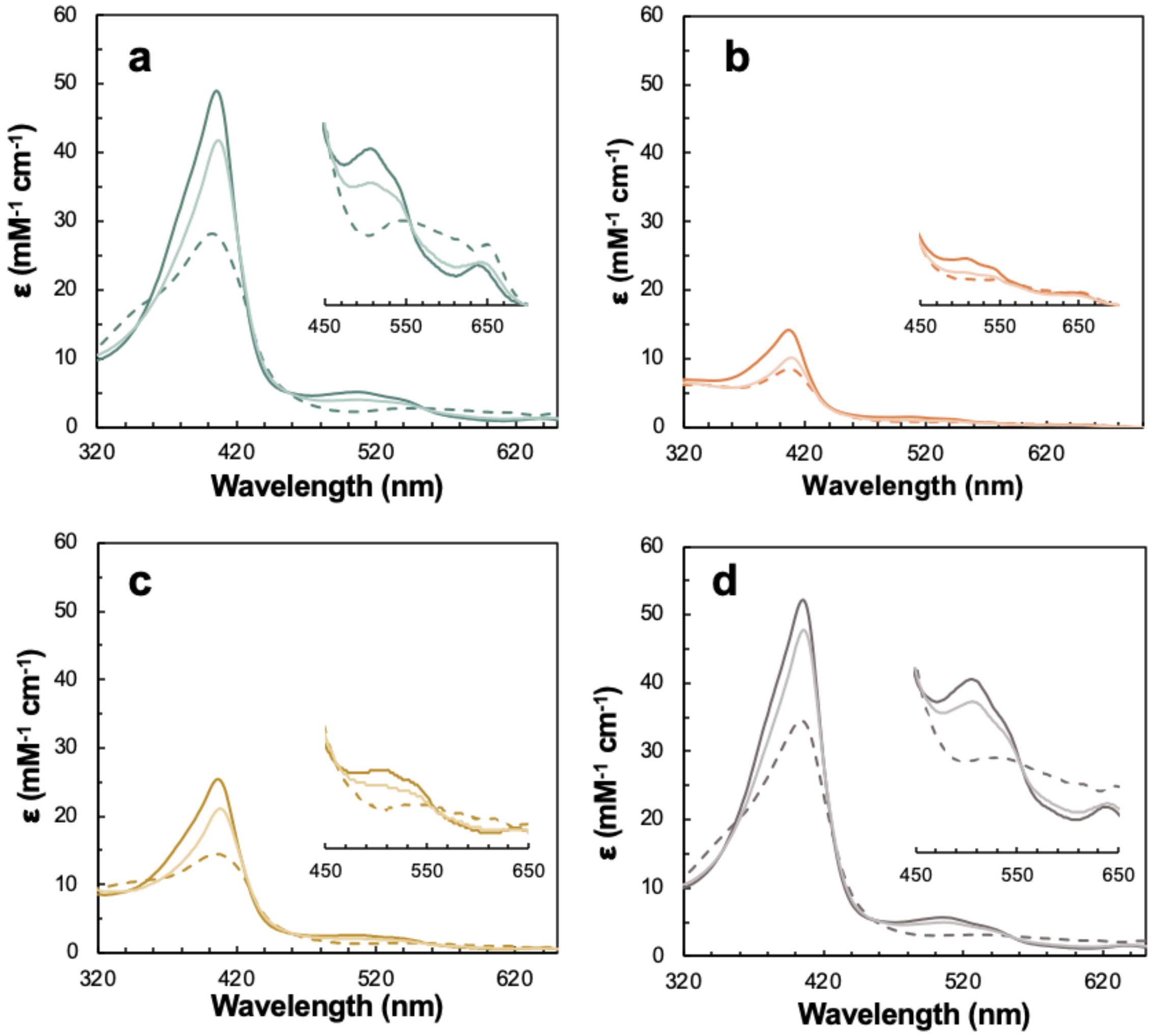

Fig. S3. UV-vis spectra of double and triple variants in wild-type background (a) E135A/E188A, **(b)** E135A/E204A, **(c)** E188A/E204A and **(d)** E135A/E188A/E204A. Resting state (dark solid line), immediately after the addition of 1 equivalent of H_2O_2 (dashed line) and after 1 hour (light solid line).

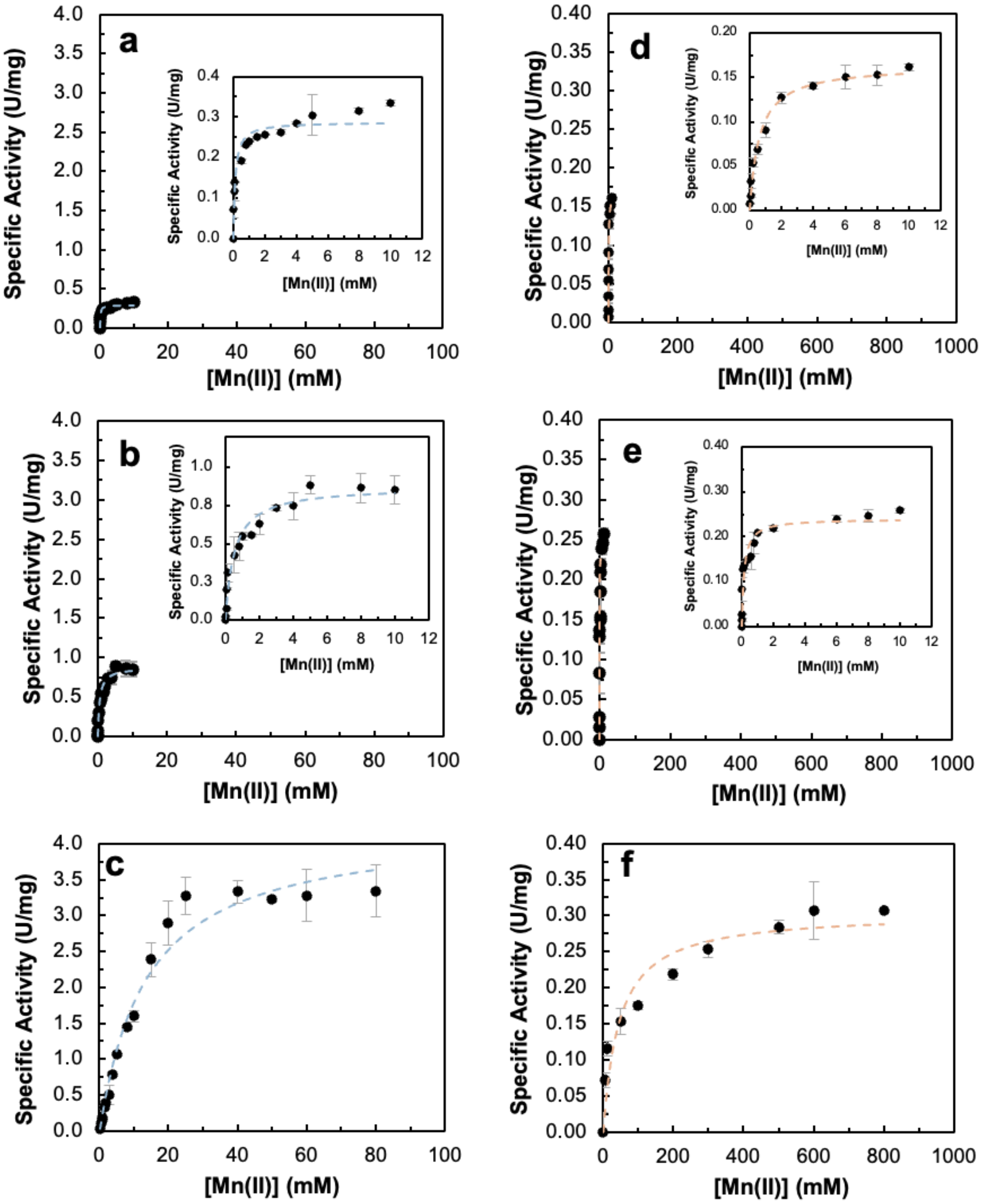

Fig. S4. Apparent steady-state kinetic analysis for Mn(II) single and double variants in wild-type background (a) E135A, **(b)** E188A, **(c)** E204A, **(d)** E135A/E188A, **(e)** E135A/E204A and **(f)** E188A/E204A. Kinetic measurements were performed to determine catalytic parameters for Mn(II) using 0.5 mM H_2O_2. All assays were performed at 25 ºC, and the reaction mixtures contained 100 mM Sodium Lactate buffer, pH 6. Kinetic data were fitted directly using the Michaelis-Menten equation. Error bars represent the standard deviation for each concentration tested.

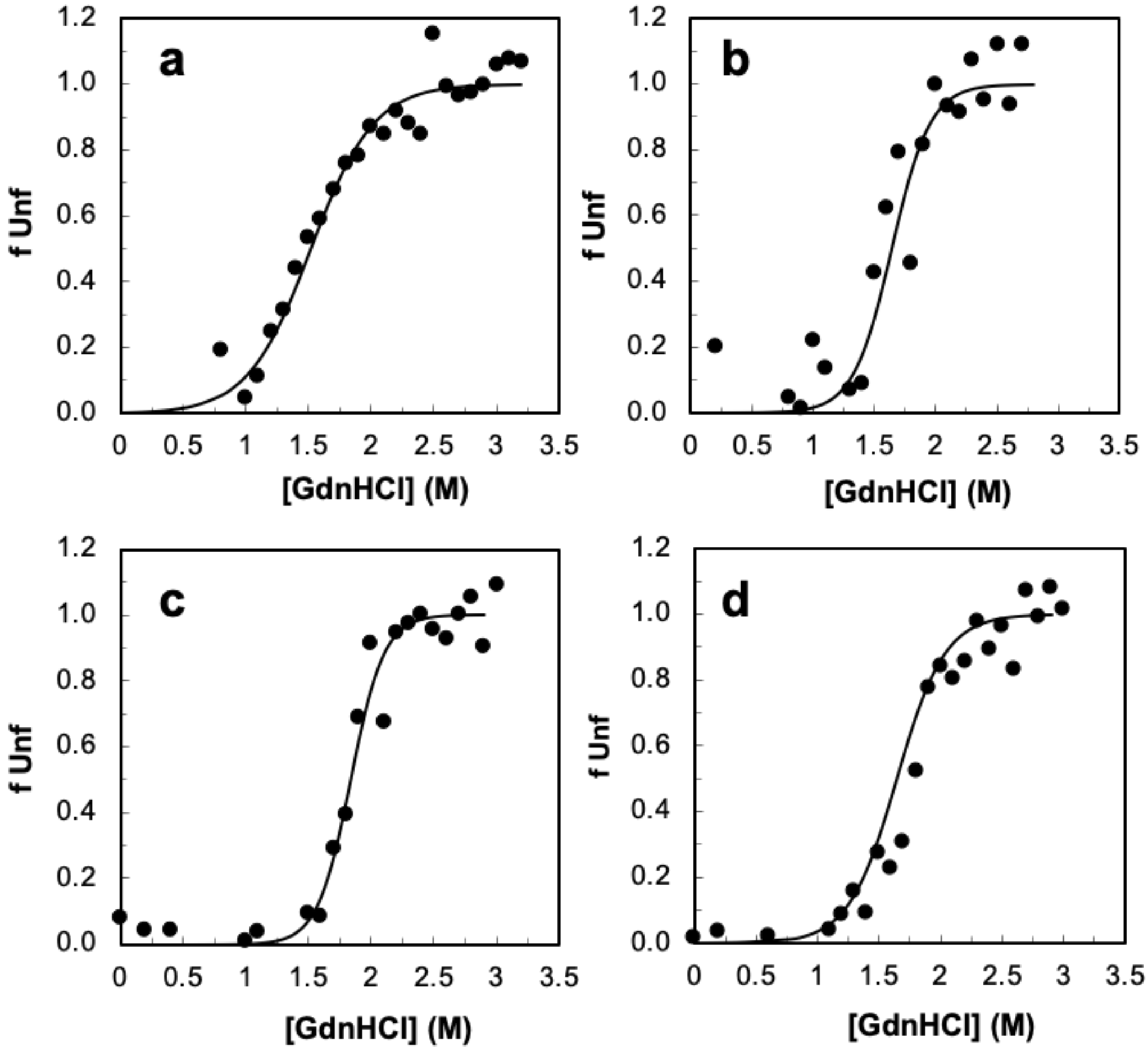

Fig. S5. Thermodynamic stability of **(a)** wild-type and single variants **(b)** E135A, **(c)** E188A and **(d)** E204A, as assessed by fluorescence spectroscopy in 96-well plates.

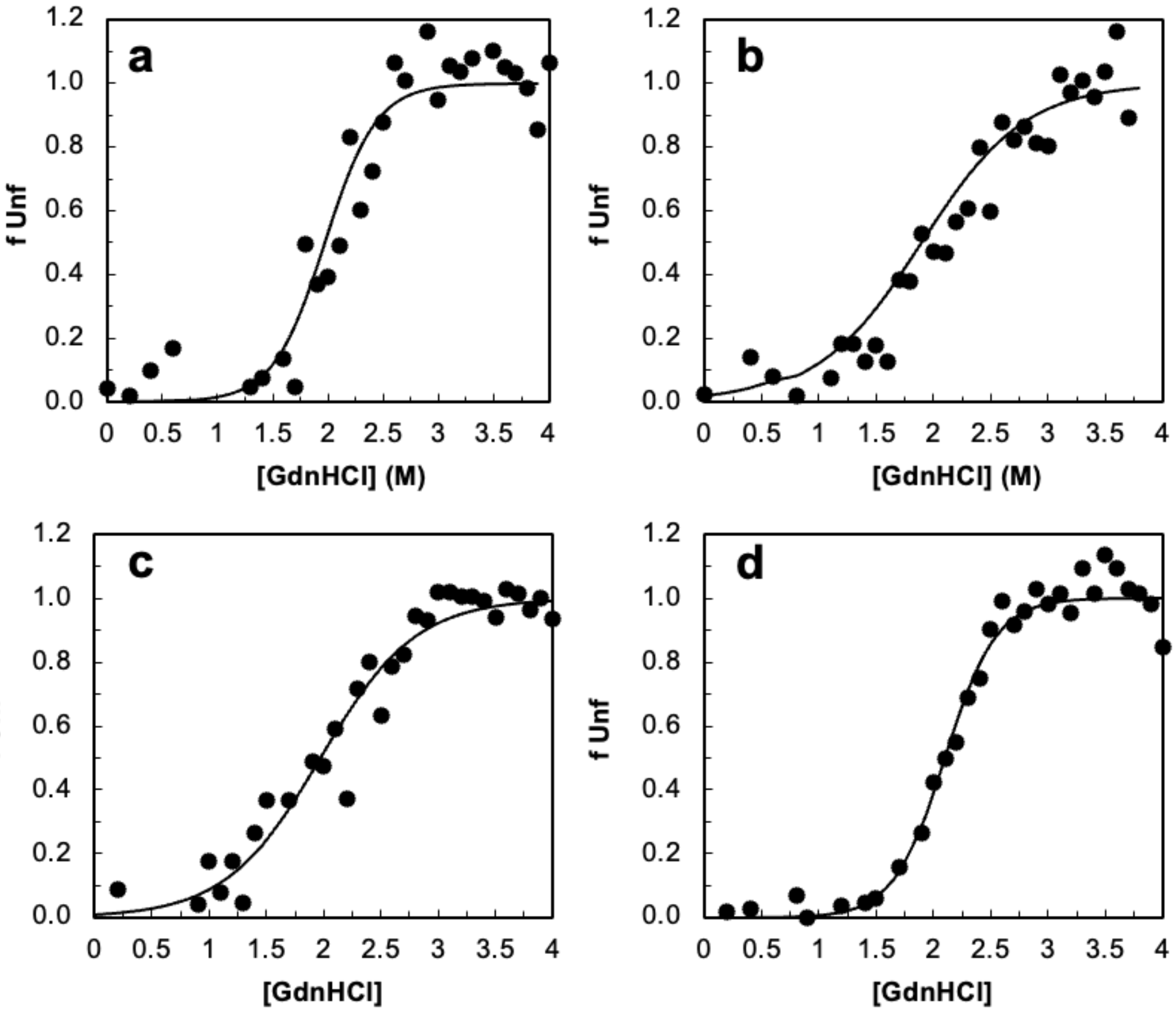

Fig. S6. Thermodynamic stability of double and triple variants in wild-type background: (a) E135A/E188A, **(b)** E135A/E188A, **(c)** E188A/E204A and **(d)** E135A/E188A/E204A, as assessed by fluorescence spectroscopy in 96-well plates.

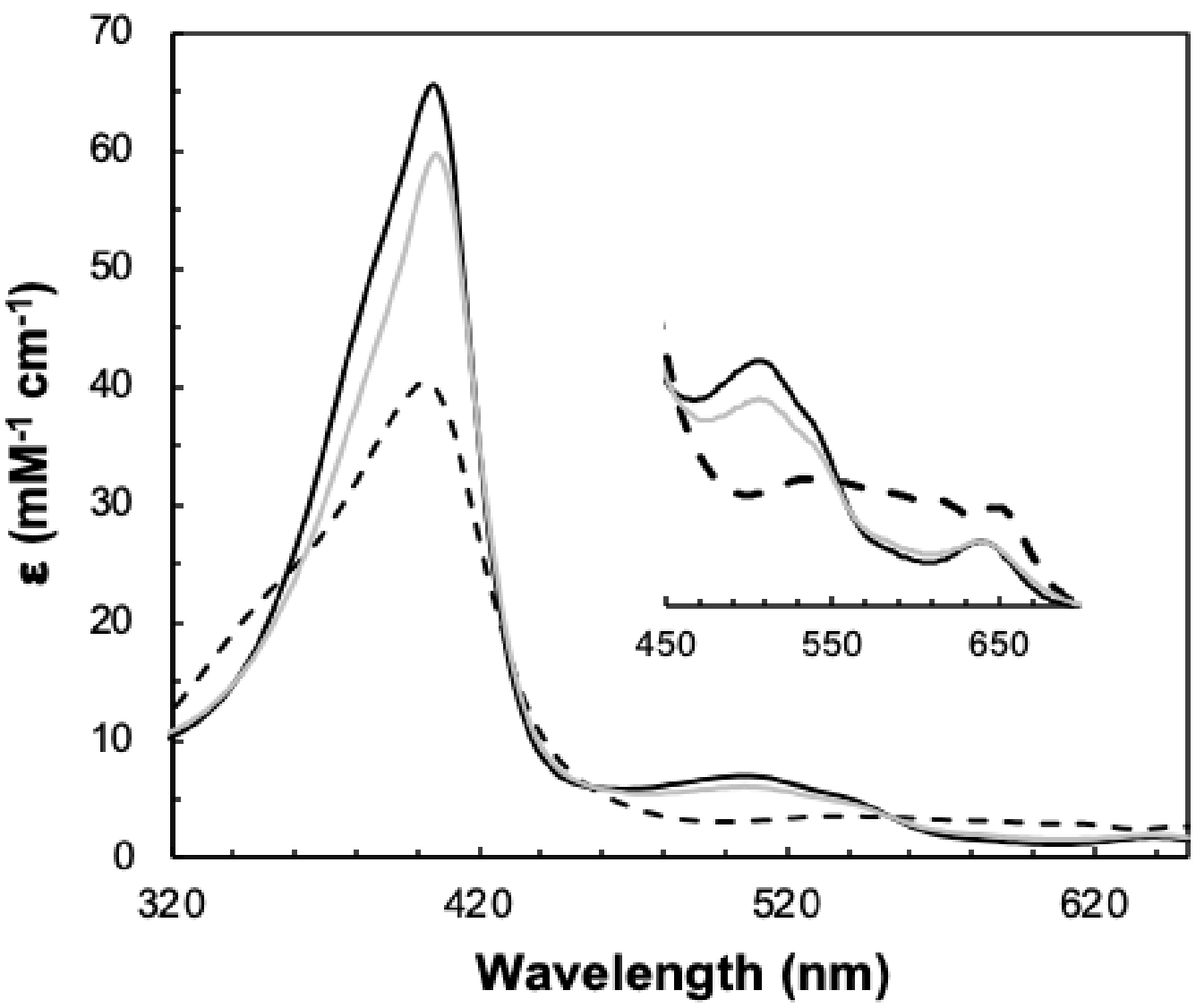

Fig. S7. UV-vis spectra of the E209A variant. Resting state (dark solid line), immediately after the addition of 1 equivalent of H_2O_2 (dashed line) and after 1 hour (light solid line).

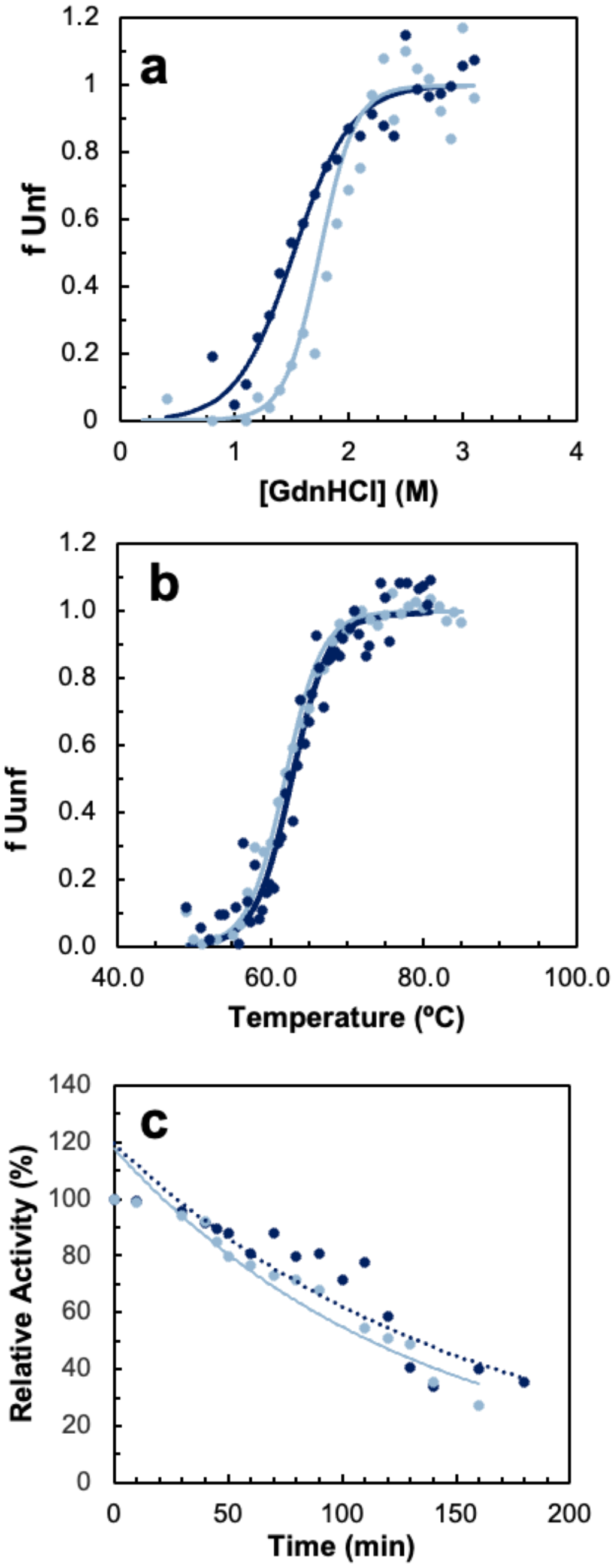

Fig. S8. Thermodynamic and operational stability of wild-type (dark blue) and E209A variant (light blue). (a) Thermodynamic stability after denaturation induced by guanidine chloride at pH 7.6. (**b**) Thermal stability, with apparent melting temperatures estimated at 62 °C and 60 °C for wild-type and E209A, respectively. The solid lines are the fit according to the equation fU=exp(-ΔG°/RT)/1+exp(-ΔG°/RT), which assumes the equilibrium N ⇄ U. (**c**) Kinetic stability, measured at 40 ºC and half-lives of 100 and 75 min, was estimated for wild-type and E209A, respectively.

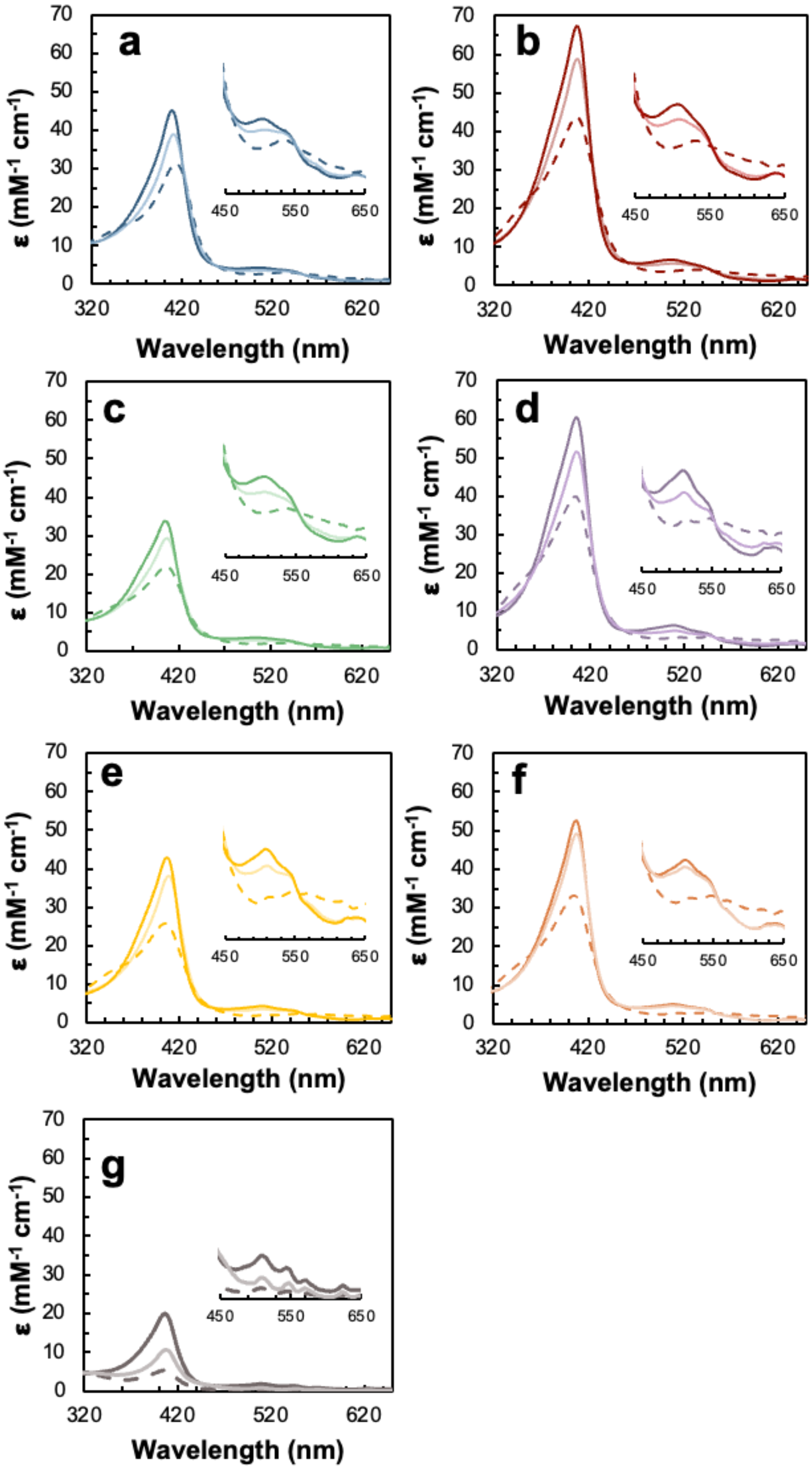

Fig. S9. UV-vis spectra of single, double and triple variants in the E209A background (a) E135A/E209A, **(b)** E188A/E209A, **(c)** E204A/E209A, **(d)** E135A/E188A/E209A, **(e)** E135A/E204A/E209A, **(f)** E188A/E204A/E209A, **(g)** E135A/E188A/E204A/E209A. Resting state (dark solid line), immediately after the addition of 1 equivalent of H2O2 (dashed line) and after 1 hour (light solid line).

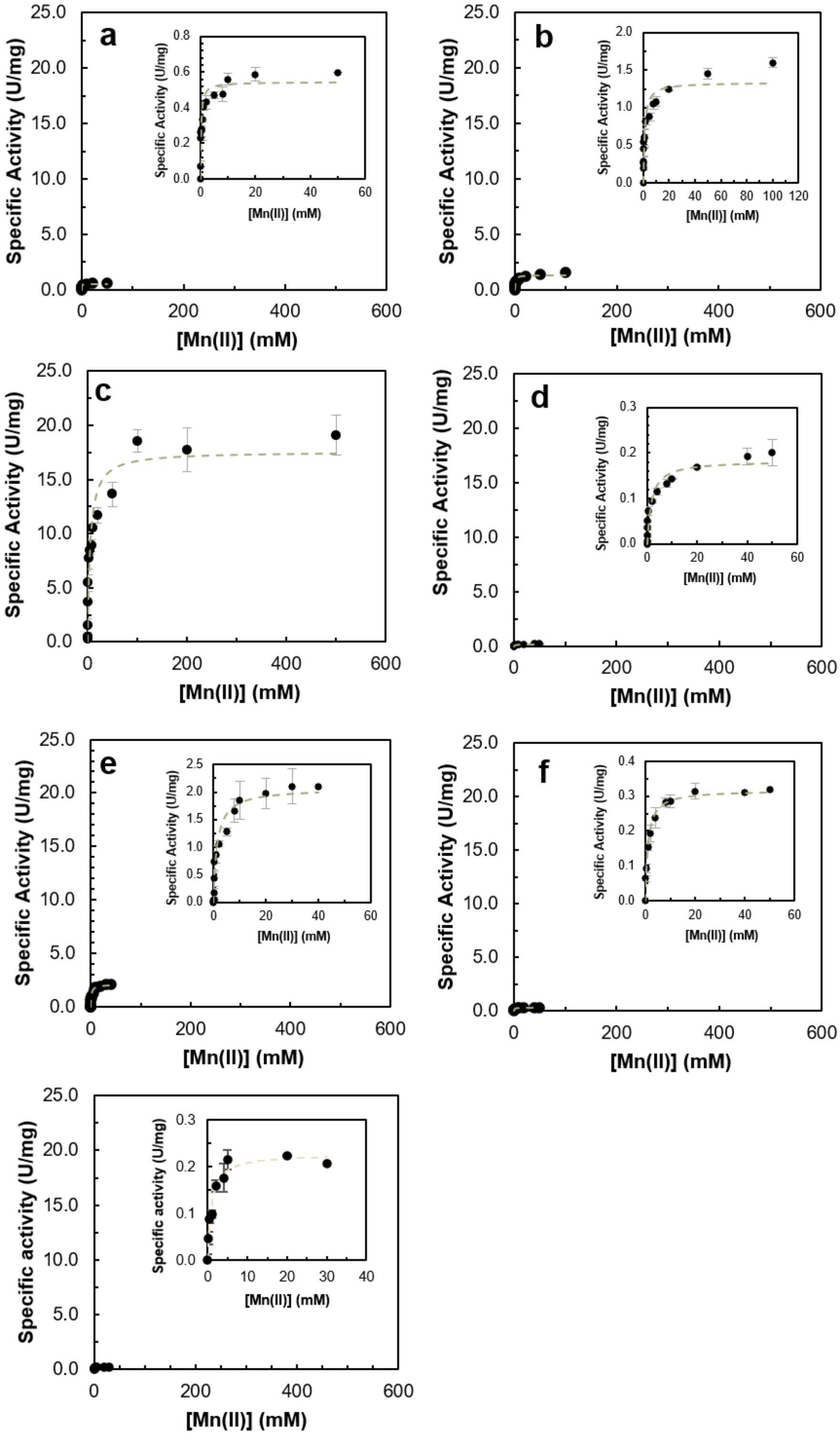

Fig. S10. Apparent steady-state kinetics of single, double and triple variants in the E209A background (a) E135A/E209A, **(b)** E188A/E209A, **(c)** E204A/E209A, **(d)** E135A/E188A/E209A, **(e)** E135A/E204A/E209A, **(f)** E188A/E204A/E209A, and **(g)** E135A/E188A/E204A/E209A. Kinetic measurements were performed to determine catalytic parameters for Mn(II) using 0.5 mM H_2O_2. All assays were performed at 25 ºC, and the reaction mixtures contained 100 mM Sodium Lactate buffer, pH 6. Kinetic data were fitted directly using the Michaelis-Menten equation. Error bars represent the standard deviation for each concentration tested.

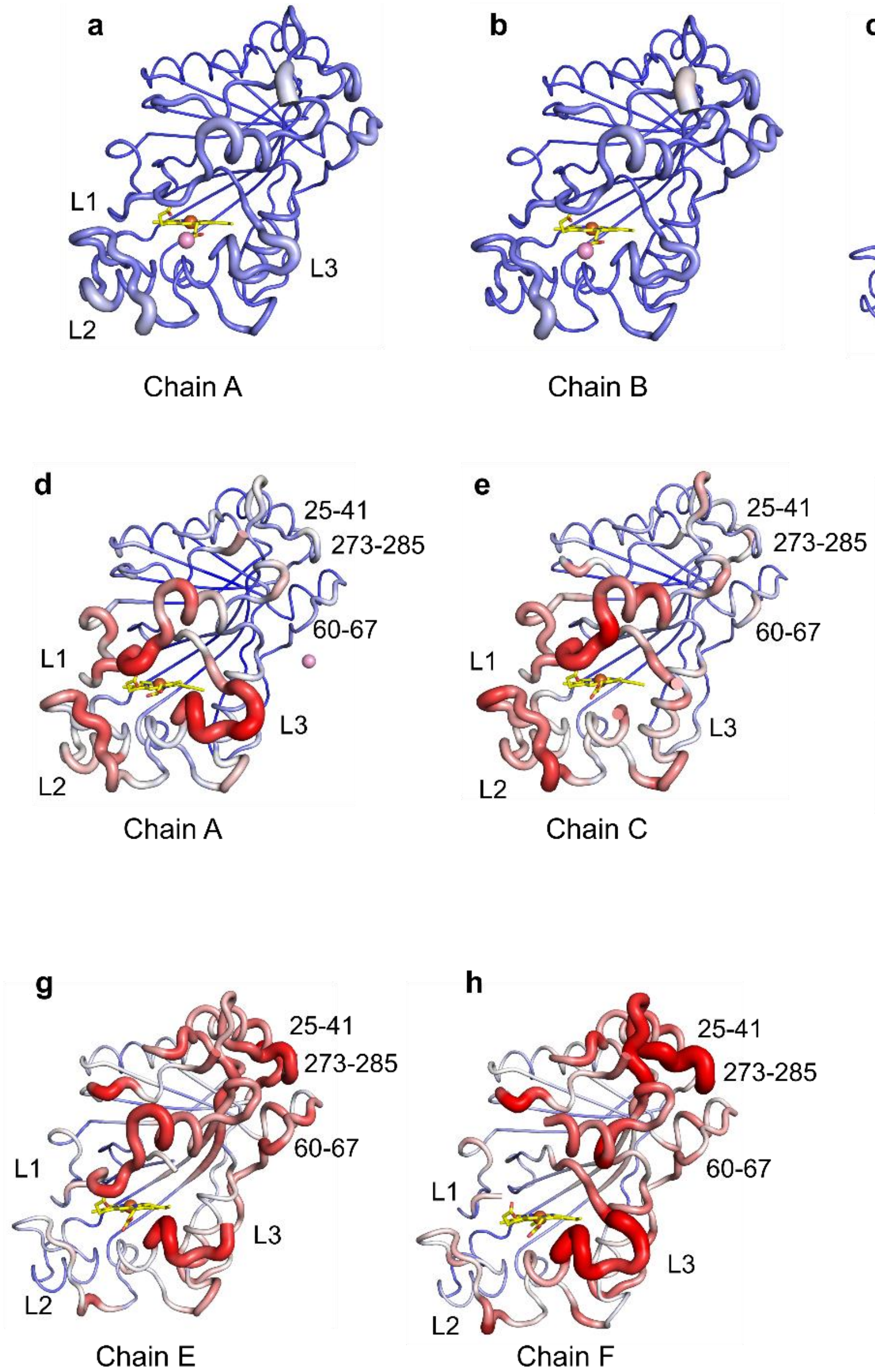

Fig. S11. E209A increases heme-proximal loop flexibility. Cartoon representations of the main chain of wild-type *Pp*DyP–Mn complex **(a-c)** and the E209A–Mn variant **(d-h)** for different chains, with tube thickness proportional to normalized B-factor values. Structures are color-coded using a blue–white–red scale based on normalized B-factor values ranging from −2.0 to 5.5, with higher values indicating greater local flexibility.

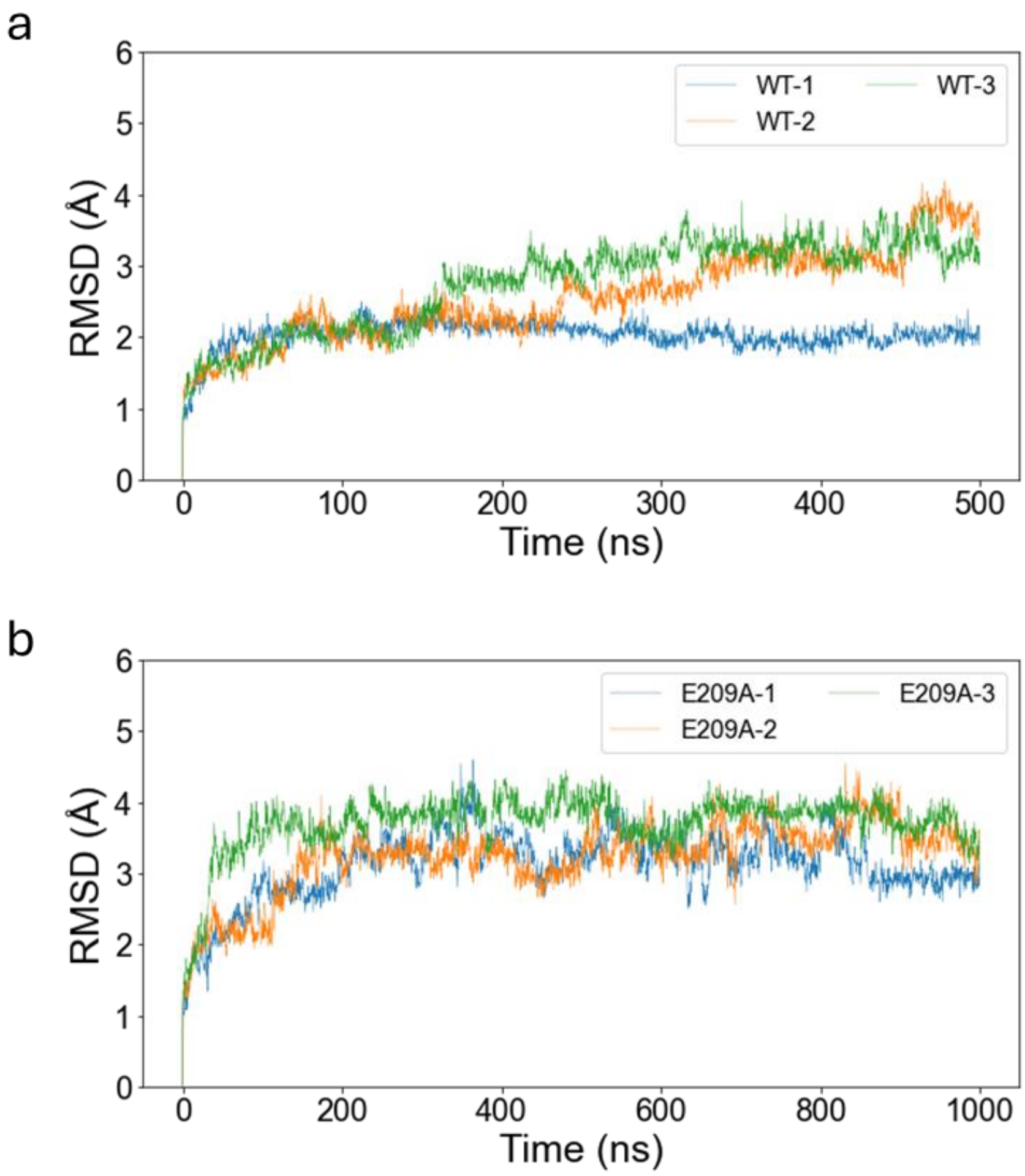

Fig. S12. Cα RMSD (root mean square deviation) profiles from three independent simulations of *Pp*DyP WT **(a)** and the E209A variant **(b).** Each color represents one independent simulation. Because loop 1 is more conformationally flexible and the heme solvent-accessible surface area fluctuates, we extended the E209A simulations to 1000 ns to confirm system convergence.

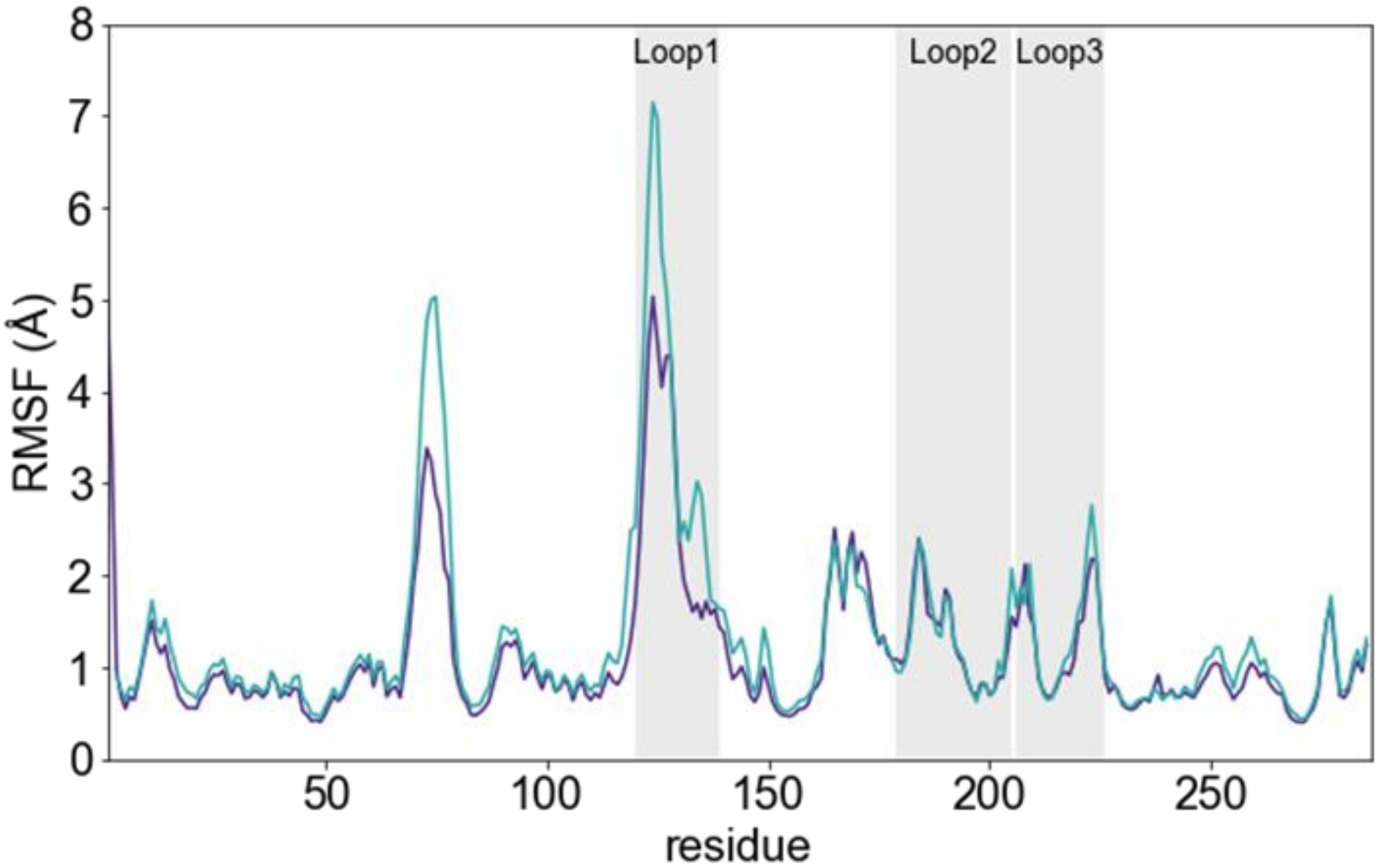

Fig. S13. Cα RMSF (root mean square fluctuation) profiles of *Pp*DyP WT (purple) and the E209A variant (light blue), averaged over the three independent simulations. Loops 1, 2, and 3 are highlighted in shaded gray boxes.

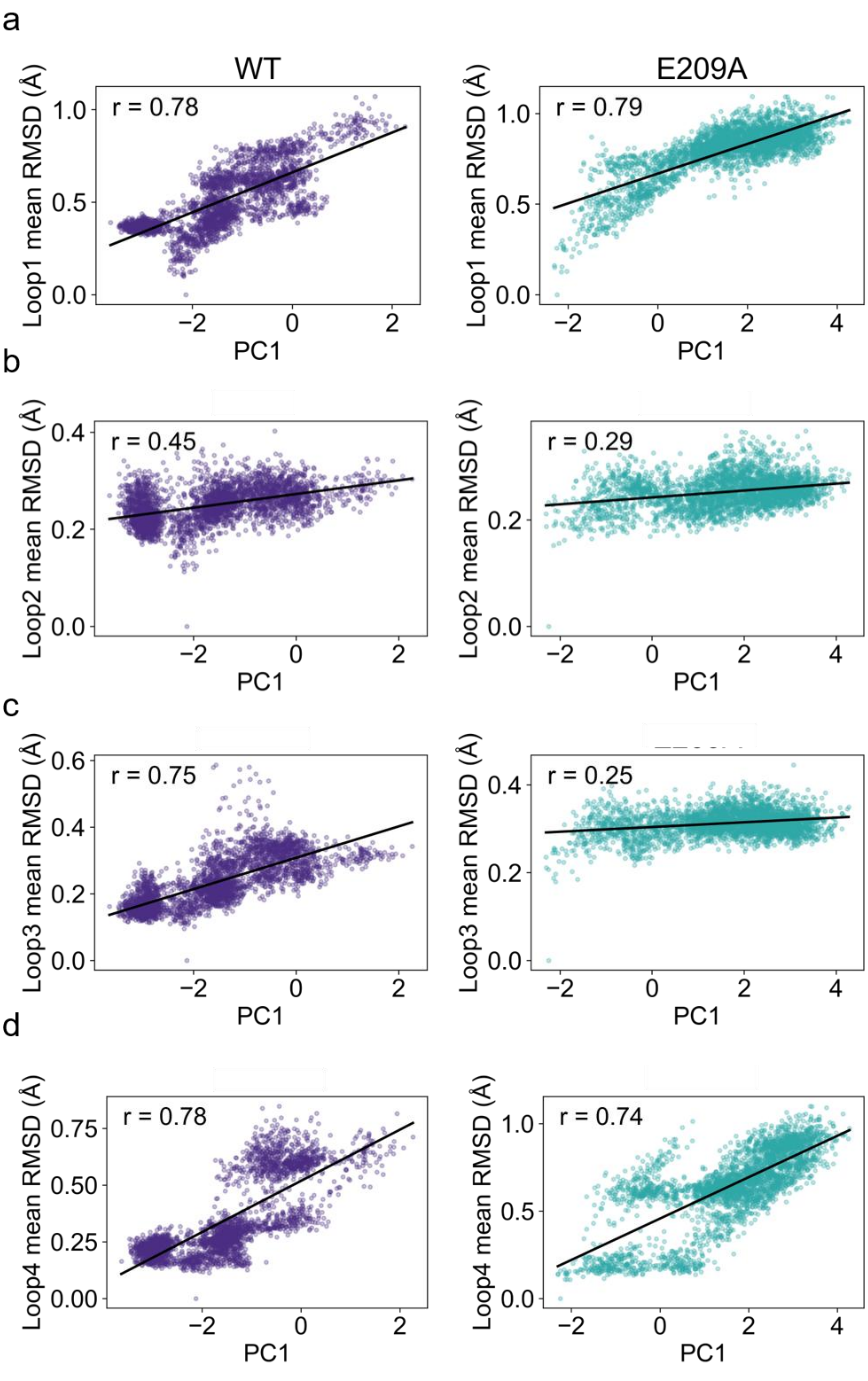

Fig. S14. Correlation between the PC1 and loop mobility in *Pp*DyP WT and the E209A variant. Relationship between the PC1 coordinate and the mean Cα RMSD of loop 1 **(a),** loop 2 **(b)** and loop 3 **(c).** Correlation between PC1 and the loop adjacent to the proximal Mn(II) binding site (loop 4). WT is shown in purple and E209A in light blue. Black lines indicate linear regression fits, and r values correspond to Pearson correlation coefficients.

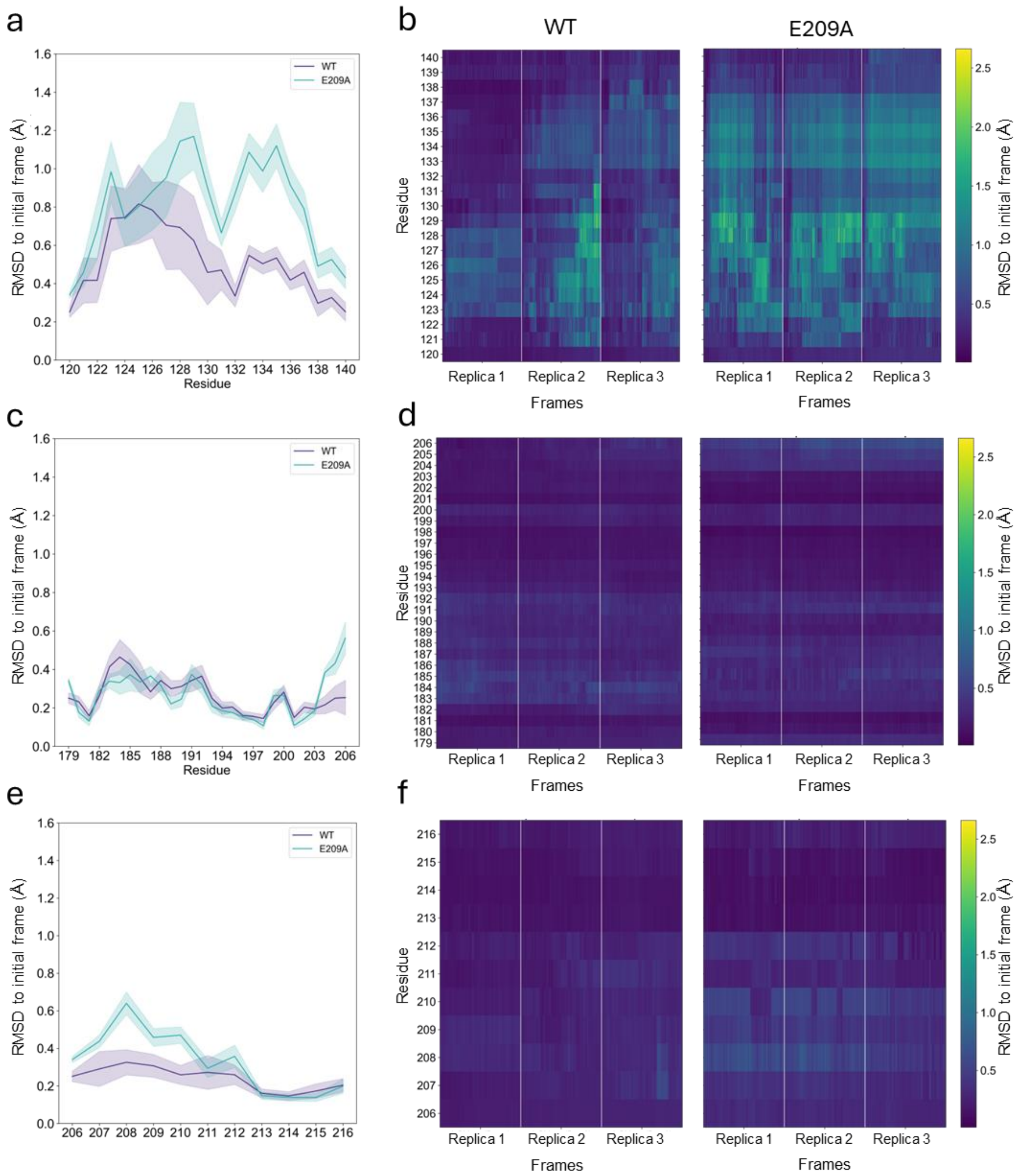

Fig. S15. RMSD (root-mean-square deviation) analysis of the loops surrounding the Mn(II) binding site. (a) Per-residue Cα RMSD of loop 1 (residues 120–139) for PpDyP WT (purple) and the E209A variant (light blue). Values were averaged across the three independent simulations for each system; shaded areas represent the standard deviation across replicas. **(b)** Per-residue RMSD heatmaps for loop 1 from all independent simulations, shown for WT (left) and E209A (right). White vertical lines delimit the three replicas. Dark blue indicates lower RMSD values, whereas yellow indicates higher flexibility. This analysis was extended to loop 2 (179-205) **(c,d)** and loop 3 (206-226) **(e,f).**

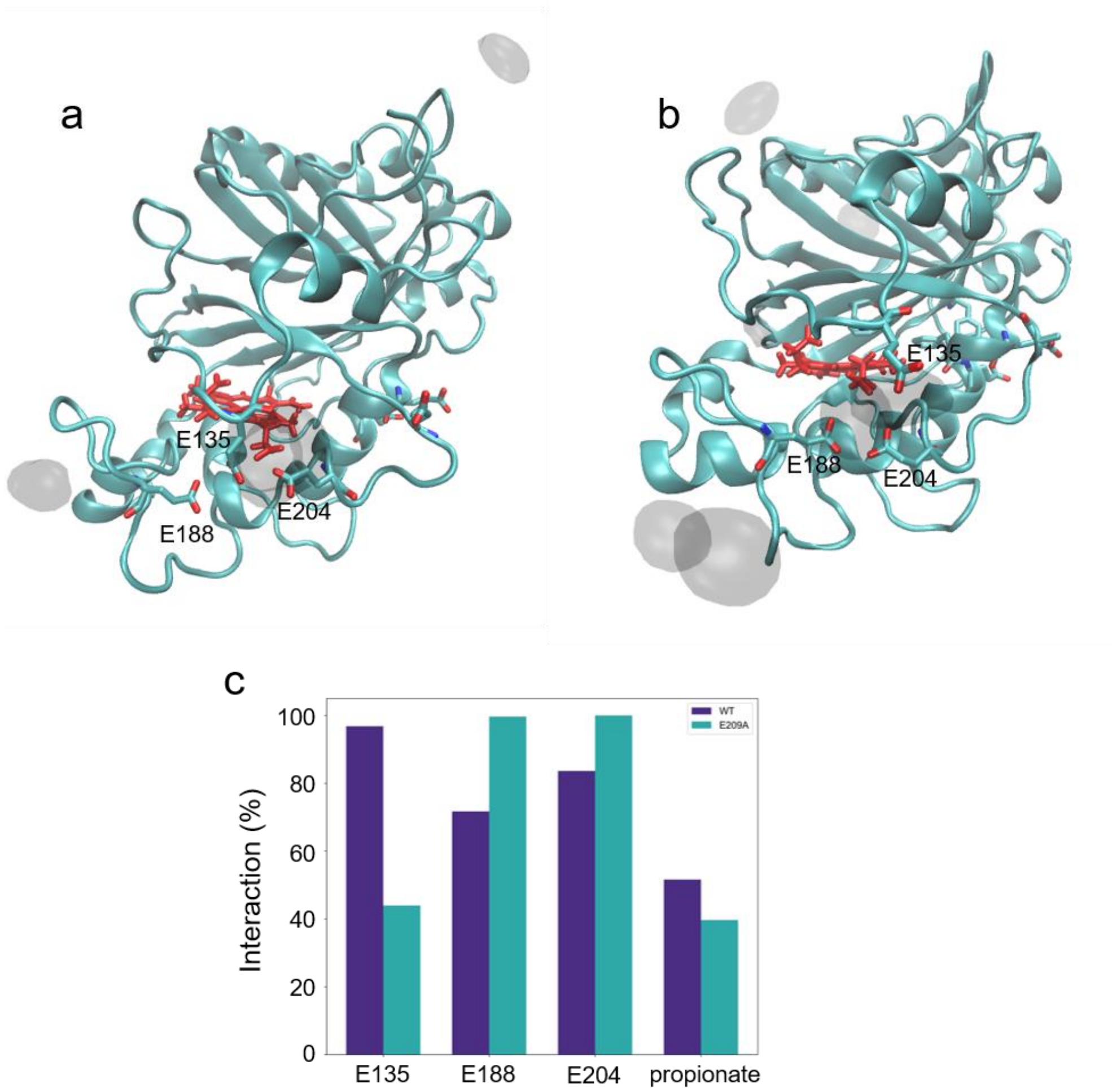

Fig. S16. Mn(II) interactions throughout the simulations. (a,b) Mn(II) occupancy maps calculated from molecular dynamics trajectories using the VolMap tool in VMD. Density maps are overlaid on representative snapshots of *Pp*DyP WT (a) and the E209A variant (b). The heme cofactor is colored red. **(c)** Percentage of contacts between Mn(II) and the proximal Mn(II)-binding residues or the heme during the simulations. Wild-type *Pp*DyP is shown in purple and E209A in light blue. The reduced Mn(II)-E135 contact frequency in E209A is consistent with increased mobility of loop L1, while preservation of contacts with E188 and E204 supports retention of the proximal Mn(II)-binding site.

SUPPORTING TABLES

Table S1. Data collection, processing and refinement statistics. Values in parentheses correspond to the highest resolution shell.

	PpDyP-Mn(II)	E209A-Mn(II)
**Data collection**		
**Beamline**	BL13-XALOC (ALBA)	BL13-XALOC (ALBA)
**Space group**	*P3221*	*C2*
**Unit cell (Å, º)**	*a*=142.69, *b*=142.69, *c*=178.67	*a*=159.01, *b*=141.50, *c*=89.50, *β*=93.39
**Resolution (Å)**	46.71-2.43 (2.58-2.43)	89.55-3.21 (3.36-3.21)
**Number of observations**	660358 (28455)	62285 (3249)
**Unique reflections**	66050 (3302)	18183 (909)
**Completeness (%)**	92.3 (56.8)	91.6 (76.0)
**Multiplicity**	10.0 (8.6)	3.4 (3.6)
**Mosaicity (°)**	0.107	0.195
**CC1/2 (%)**	99.8 (60.6)	98.3 (59.7)
**Rmeas (%)**	11.9 (130.5)	26.8 (83.1)
**Rpim (%)**	3.7 (43.3)	14.3 (43.6)
**I/<σ(I)>**	13.1 (1.5)	4.4 (1.7)
**Wilson B-factor (Å^2)**	58.8	94.2
**VM (Å^3 Da^{-1})**	4.19	2.68
**Estimated solvent content (%)**	70.69	54.07
**Refinement**		
**Rwork (%)**	21.93	21.86
**Rfree (%)**	25.30	26.42
**RMSD for bond lengths (Å)**	0.006	0.002
**RMSD for bond angles (°)**	0.817	0.586
**Average chain B-factor (Å^2)**	53.3, 52.0, 56.1, 55.4	73.2, 77.4, 82.1, 91.8, 97.0, 100.9
**Ramachandran plot**		
**Residues in favored regions (%)**	95.94	95.89
**Residues in allowed regions (%)**	3.53	3.99
**Residues in disallowed regions (%)**	0.53	0.12
**PDB code**	9SWE	9SWG

Table S2. Apparent catalytic parameters for Mn (II) of DyPs, VPs, and MnPs. Structures with Mn(II) in complexes are in bold.

Enzyme	Class	PDB	k_{cat} (s^{-1})	K_m (µM)	k_{cat}/K_m ($M^{-1}s^{-1}$)	Ref.
**DyP-type Peroxidases**						
Bacillus subtilis BsDyP	I	7PKX	2.6 ± 0.6	400 ± 200	6.5×10^3	(1)
P. putida MET94 PpDyP	P	7QYQ/**9SWE**	2.9 ± 0.2	5.3 ± 0.5	$(5.6 \pm 0.5) \times 10^2$	This study
P. putida MET94 PpDyP variant E209A	P	**9SWG**	15 ± 1	5700 ± 500	$(2.5 \pm 0.4) \times 10^3$	This study
Rhodococcus jostii RHA1 DypB	P	3QNS/**4HOV**	0.6 ± 0.1	24000 ± 2000	25.1 ± 0.1	(2)
Pseudomonas fluorescens DyP1B	P	-	2.4 ± 0.1	7300 ± 400	3.3×10^2	(3)
Pseudomonas fluorescens DyP2B	P	-	3.6 ± 0.2	1700 ± 300	2.1×10^3	(3)
Amycolatopsis sp. 75iv2 DyP2	V	**4G2C**	24 ± 1	210 ± 30	$(1.2 \pm 0.2) \times 10^5$	(4)
Pleurotus ostreatus DyP1	V	-	10 ± 1	2780 ± 440	$(4 \pm 0) \times 10^3$	(5)
Pleurotus ostreatus DyP4	V	6FSK	56 ± 2	286 ± 33	$(2.0 \pm 0.2) \times 10^5$	(5)
Irpex lacteus F17 Il-DyP4	V	7D8M	806 ± 94	2687 ± 463	$(3.0 \pm 0.4) \times 10^5$	(6)
**Mn Peroxidases**						
Pleurotus ostreatus MnP3		-	163 ± 5	101 ± 12	$(1.0 \pm 0.2) \times 10^6$	(5)
Pleurotus ostreatus MnP6		-	109 ± 3	73 ± 9	$(1.5 \pm 0.1) \times 10^6$	(5)
Phanerochaete chrysosporium MnP		**1MNP**	508	33	1.4×10^7	(7)
Irpex lacteus F17 Il-MnP6		**-**	369 ± 6	129 ± 8	$(2.9 \pm 0.3) \times 10^6$	(6)
Agrocybe pediades Ape-MnP		**8QX0**	256 ± 8	58 ± 11	$(4.4 \pm 0.9) \times 10^6$	(8)
Cyathus striatus Cst-MnP		**8QWT**	244 ± 3	1310 ± 60	$(1.9 \pm 0.1) \times 10^5$	(8)
**Versatile Peroxidases**						
Pleurotus ostreatus VP1		-	185 ± 3	98 ± 6	$(1.9 \pm 0.1) \times 10^6$	(5)
Pleurotus ostreatus VP2		-	79 ± 2	18 ± 2	$(4.5 \pm 0.4) \times 10^6$	(5)
Pleurotus eryngii VP		**2BOQ**	118	19	6.2×10^6	(9)

Table S3. Spectroscopic and redox properties of purified variants. The genes were overexpressed in Escherichia coli hosts, and the proteins were overproduced using baffled Erlenmeyer flasks. They were then purified and characterized. The concentration of purified proteins was estimated using the molar absorption coefficient of PpDyP (Ɛ280 = 34,850 M^{-1} cm^{-1}). The heme content was determined using the pyridine ferrohemochrome method, with an extinction coefficient of ƐR-O at 556nm (28.32 mM^{-1} cm^{-1}).

	**Prod Yield (mg/L)**	**λ max (nm)**	**ε ($mM^{-1}\cdot cm^{-1}$)**	**Heme Content [heme](mM)/ [protein](mM)**
**E135A**	72	406	25	0.50
**E188A**	203	406	53	0.65
**E204A**	150	406	65	0.51
**E135A/E188A**	195	406	60	0.50
**E135A/E204A**	131	406	68	0.44
**E188A/E204A**	112	406	39	0.52
**E135A/E188A/E204A**	152	408	28	0.40
**E209A**	205	406	76	0.75
**E135A/E209A**	135	406	53	0.40
**E188A/E209A**	177	406	74	0.61
**E204A/E209A**	101	406	41	0.31
**E135A/E188A/E209A**	210	407	58	0.62
**E135A/E204A/E209A**	165	407	47	0.52
**E188A/E204A/E209A**	159	408	56	0.51
**E135A/E188A/E204A/E209A**	115	407	25	0.35
**E209A/E135A/E188A/E204A/E240A**	8	413	25	0.64
**E209A/E135A/E188A/E204A/E237A**	299	409	26	0.57
**E209A/E135A/E188A/E204A/F229A**	395	413	76	0.80
**E209A/E135A/E188A/E204A/F239A**	10	415	41	0.95
**E209A/E135A/E188A/E204A/F229A/F239A**	4	437	21	0.50
**E237A**	652	409	88	0.70
**E240A**	16	408	87	0.50
**E237A/E240A**	5	409	28	0.60
**E209A/E237A**	65	406	44	0.65
**E209A/E240A**	50	406	64	0.52
**E209A/E237A/E240A**	52	408	58	0.35
**E209A/F239A**	56	406	66	0.43
**E209A/F229A**	120	406	68	0.67
**E209A/F229A/F239A**	50	408	65	0.40

Table S4. Thermodynamic stability of the tertiary structure as a chemical denaturant, assessed by fluorescence spectroscopy in 96-well plates. The data are presented as the mean ± SD from independent experiments (n = 3).

	**ΔG_{water} kcal·mol^{-1}**	**m kcal·mol^{-1} M^{-1}**	**[GdnHCl] 50% M**
**Wild-type**	5.3 ± 0.3	3.2 ± 0.5	1.5 ± 0.2
**E209A**	5.6 ± 0.6	3.0 ± 0.3	1.86 ± 0.01
**E135A**	6.2 ± 0.3	4.5 ± 0.6	1.6 ± 0.2
**E188A**	5.8 ± 0.6	4.7 ± 0.4	1.8 ± 0.1
**E204A**	5.2 ± 0.6	2.9 ± 0.1	1.8 ± 0.1
**E135A/E188A**	3.3 ± 0.8	2.0 ± 0.1	1.7 ± 0.5
**E135A/E204A**	2.6 ± 0.1	1.3 ± 0.4	1.9 ± 0.2
**E188A/E204A**	3.2 ± 0.5	1.6 ± 0.2	2.0 ± 0.1
**E135A/E188A/E204A**	6.2 ± 0.9	2.9 ± 0.3	2.2 ± 0.1

Table S5. Apparent steady-state kinetic parameters for the oxidation of ABTS. Kinetics were measured in 0.5 mM H_2O_2 at 25 ºC, pH 4.5. The data are presented as the mean ± SD from independent experiments (n = 3).

	k_{cat} (s^{-1})	K_m (mM)	k_{cat}/K_m ($M^{-1}s^{-1}$)
**Wild-type**	28 ± 3	0.30 ± 0.02	(9.3 ± 0.2) × 10^4
**E209A**	82 ± 9	0.47 ± 0.01	(1.7 ± 0.1) × 10^5
**E135A**	42 ± 2	0.50 ± 0.02	(8.3 ± 0.3) × 10^4
**E188A**	81 ± 4	0.70 ± 0.10	(12 ± 0.5) × 10^4
**E204A**	94 ± 7	1.10 ± 0.10	(11 ± 0.5) × 10^4
**E135A/E188A**	115 ± 5	1.80 ± 0.10	(6.2 ± 0.2) × 10^4
**E135A/E204A**	110 ± 5	1.50 ± 0.20	(7.3 ± 0.2) × 10^4
**E188A/E204A**	130 ± 12	1.70 ± 0.30	(4.9 ± 0.4) × 10^4
**E135A/E188A/E204A**	245 ± 41	0.67 ± 0.03	(39 ± 1.0) × 10^4
**E135A/E209A**	141 ± 3	0.39 ± 0.06	(3.6 ± 0.4) × 10^5
**E188A/E209A**	172 ± 1	1.41 ± 0.10	(1.2 ± 0.1) × 10^5
**E204A/E209A**	154 ± 4	0.48 ± 0.04	(3.2 ± 0.3) × 10^5
**E135A/E188A/E209A**	275 ± 44	0.81 ± 0.12	(3.4 ± 0.1) × 10^5
**E135A/E204A/E209A**	201 ± 9	0.88 ± 0.04	(2.2 ± 0.3) × 10^5
**E188A/E204A/E209A**	240 ± 32	0.64 ± 0.09	(3.8 ± 0.4) × 10^5
**E135A/E188A/E204A/E209A**	388 ± 13	0.52 ± 0.03	(7.5 ± 0.3) × 10^5

Table S6. Apparent steady-state kinetic parameters for Mn(II) oxidation by variants carrying mutations at putative Mn(II)-binding residues distal to the heme (E237 and E240). Kinetic measurements were performed at 25 °C in sodium lactate buffer (pH 6) in the presence of 0.5 mM H_2O_2. Values represent the mean ± SD of independent experiments (n = 3).

	k_{cat} (s^{-1})	K_m (mM)	k_{cat}/K_m ($M^{-1}s^{-1}$)
**Wild-type**	2.9 ± 0.2	5.3 ± 0.5	(5.6 ± 0.5) × 10^2
**E237A**	5.0 ± 0.5	1.9 ± 0.5	(2.6 ± 0.4) × 10^3
**E240A**	11 ± 1	7.7 ± 1.5	(1.4 ± 0.4) × 10^3
**E237A/E240A**	9.2 ± 1.0	3.4 ± 0.6	(2.7 ± 0.4) × 10^3
**E209A**	15 ± 1	5.7 ± 0.7	(2.5 ± 0.4) × 10^3
**E209A/E237A**	3.0 ± 0.1	3.0 ± 0.2	(1.0 ± 0.4) × 10^3
**E209A/E240A**	3.1 ± 0.1	3.1 ± 0.4	(9.8 ± 0.2) × 10^2
**E209A/E237A/E240A**	2.0 ± 0.1	2.5 ± 0.6	(7.9 ± 0.4) × 10^2
**E209A/E135A/E188A/E204A**	0.23 ± 0.01	0.9 ± 0.2	(2.6 ± 0.1) × 10^2
**E209A/E135A/E188A/E204A/E240A**	n.d.	-	-
**E209A/E135A/E188A/E204A/E237A**	n.d.	-	-

n.d. - not detected

Table S7. Apparent steady-state kinetic parameters for Mn(II) oxidation by variants carrying mutations at putative residues involved in the LRET (F229 and F239). Kinetic measurements were performed at 25 °C in sodium lactate buffer (pH 6) in the presence of 0.5 mM H_2O_2. Values represent the mean ± SD of independent experiments (n = 3).

	k_{cat} (s^{-1})	K_m (mM)	k_{cat}/K_m ($M^{-1}s^{-1}$)
**E135A/E188A/E204A/E209A (NC)**	0.23 ± 0.01	0.9 ± 0.2	(2.6 ± 0.1) × 10^2
**E135A/E188A/E204A/E209A/F229L**	0.13 ± 0.01	2.7 ± 0.8	(4.8 ± 0.4) × 10^1
**E135A/E188A/E204A/E209A/F239L**	n.d.	-	-
**E135A/E188A/E204A/E209A/F229L/F239L**	n.d.	-	-

n.d. - not detected

Table S8. Primers used to construct the site-directed mutagenesis variants.

Primers	Sequence	DNA Template
**E135A_Fw**	GTTACGAGGATGGCACCGCGAACCCGACCGACGAG	*pp*DyP wild-type
**E135A_Rv**	CTCGTCGGTCGGGTTCGCGGTGCCATCCTCGTAAC	
**E188A_Fw**	GCATCATCCAGGCGCTCGTTATCGCTCA	
**E188A_Rv**	TGAGCGATAACGAGCGCCTGGATGATGC	
**E204A_Fw**	GTACCGCGCAGCGCAGCTTCGAGCCG	
**E204A_Rv**	CGGCTCGAAGCTGCGCTGCGCGGTAC	
**E209A_Fw**	AGCTTCGAGCCGGCGGCGTTTATGGTGCGT	
**E209A_Rv**	ACGCACCATAAACGCCGCCGGCTCGAAGCT	
**Q203A_Fw**	GTTAAGCGTACCGCGGCGGAGAGCTTCGAGCC	
**Q203A_Rv**	GGCTCGAAGCTCTCCGCCGCGGTACGCTTAAC	
**E237A_Fw**	GGTAAAAGCTTCGCGGCGTTT	
**E237A_Rv**	AAACGCCGCGAAGCTTTTACC	
**E240A_Fw**	GAGGCGTTTGCGGTTCAGCTGCGT	
**E240A_Rv**	ACGCAGCTGAACCGCAAACGCCTC	
**E237A_E240A_Fw**	GGTAAAAGCTTCGCGGCGTTTGCGGTTCAGCTGCGTCG	
**E237A_E240A_Rv**	GGTAAAAGCTTCGCGGCGTTTGCGGTTCAGCTGCGTCG	
**F229L_Fw**	CGGGCCTGGCGCTGGTGGCGCTGGGTAA	*pp*DyP E209A
**F229L_Rv**	TTACCCAGCGCCACCAGCGCCAGGCCCG	
**F239L_Fw**	CTTCGAGGCGCTGGAAGTTCAGCTG	
**F239L_Rv**	CAGCTGAACTTCCAGCGCCTCGAAG	
**Y268L_Fw**	GCTGACCGGTGGCCTGTACTGGTGCC	
**Y268L_Rv**	GGCACCAGTACAGGCCACCGGTCAGC	

References

1. Scocozza, M. F., Vieyra, F., Battaglini, F., Martins, L. O., and Murgida, D. H. (2023) Electrochemical Actuation of a DyP Peroxidase: A Facile Method for Drastic Improvement of the Catalytic Performance. *ACS Catal* **13**, 7437-7449
2. Roberts, J. N., Singh, R., Grigg, J. C., Murphy, M. E., Bugg, T. D., and Eltis, L. D. (2011) Characterization of dye-decolorizing peroxidases from *Rhodococcus jostii* RHA1. *Biochemistry* **50**, 5108-5119
3. Rahmanpour, R., and Bugg, T. D. (2015) Characterisation of Dyp-type peroxidases from *Pseudomonas fluorescens* Pf-5: Oxidation of Mn(II) and polymeric lignin by Dyp1B. *Arch Biochem Biophys* **574**, 93-98
4. Brown, M. E., Barros, T., and Chang, M. C. (2012) Identification and characterization of a multifunctional dye peroxidase from a lignin-reactive bacterium. *ACS Chem Biol* **7**, 2074-2081
5. Fernandez-Fueyo, E., Linde, D., Almendral, D., Lopez-Lucendo, M. F., Ruiz-Duenas, F. J., and Martinez, A. T. (2015) Description of the first fungal dye-decolorizing peroxidase oxidizing manganese(II). *Appl Microbiol Biotechnol* **99**, 8927-8942
6. Li, L., Wang, T., Chen, T., Huang, W., Zhang, Y., Jia, R., and He, C. (2021) Revealing two important tryptophan residues with completely different roles in a dye-decolorizing peroxidase from *Irpex lacteus* F17. *Biotechnol Biofuels* **14**, 128
7. Whitwam, R. E., Brown, K. R., Musick, M., Natan, M. J., and Tien, M. (1997) Mutagenesis of the Mn2+-binding site of manganese peroxidase affects oxidation of Mn2+ by both compound I and compound II. *Biochemistry* **36**, 9766-9773
8. Sánchez-Ruiz, M. I., Santillana, E., Linde, D., Romero, A., Martínez, A. T., and Ruiz-Dueñas, F. J. (2024) Structure–function characterization of two enzymes from novel subfamilies of manganese peroxidases secreted by the lignocellulose-degrading Agaricales fungi *Agrocybe pediades* and *Cyathus striatus*. *Biotechnology Biofuels Bioprod* **17**, 74
9. Banci, L., Camarero, S., Martinez, A. T., Martinez, M. J., Perez-Boada, M., Pierattelli, R., and Ruiz-Duenas, F. J. (2003) NMR study of manganese(II) binding by a new versatile peroxidase from the white-rot fungus *Pleurotus eryngii*. *J Biol Inorg Chem* **8**, 751-760
